# Supplementary material for: Targeting HMGB2 acts as dual immunomodulator by bolstering CD8+ T cell function and inhibiting tumor growth in hepatocellular carcinoma
Source: Sci Adv. 2025 May 2;11(18):eads8597. doi: 10.1126/sciadv.ads8597 (PMC12047442; doi:10.1126/sciadv.ads8597)
Supplement: 20250502-1 [file sciadv.ads8597.v1.pdf]

CANCER

Targeting HMGB2 acts as dual immunomodulator by bolstering CD8$^+$ T cell function and inhibiting tumor growth in hepatocellular carcinoma

Wei-Feng Qu[1,2,3]†, Gui-Qi Zhu[1,2]†, Rui Yang[1,2]†, Tian-Hao Chu[1,2]†, Zhi-Qi Guan[1,2]†, Run Huang[1,2,3], Meng-Xin Tian[4], Xi-Fei Jiang[1,2], Chen-Yang Tao[1,2], Yuan Fang[1,2], Jun Gao[1,2], Xiao-Ling Wu[1,2], Jia-Feng Chen[1,2], Qian-Fu Zhao[1,2], Yi Wang[1,2], Yi-Chao Bu[1,2], Jian Zhou[1,2], Jia Fan[1,2], Wei-Ren Liu[1,2]*, Zheng Tang[1,2]*, Ying-Hong Shi[1,2]*

Copyright © 2025 The Authors, some rights reserved; exclusive licensee American Association for the Advancement of Science. No claim to original U.S. Government Works. Distributed under a Creative Commons Attribution License 4.0 (CC BY).

T cell exhaustion is a critical obstacle for durable treatment response in hepatocellular carcinoma (HCC). Developing drugs that control tumor growth and simultaneously bolster immune function is of great significance. Although high-mobility group box 2 (HMGB2) has been reported to be crucial to HCC prognosis, its role in the tumor microenvironment remains unclear. Here, we found HMGB2$^+$ CD8$^+$ T cells as being associated with immune exhaustion and resistance to anti–PD-1 treatment through single-cell RNA sequencing. Mechanistically, HMGB2 impaired the oxidative phosphorylation in CD8$^+$ T cells and inactivated the interferon-γ response in tumor cells, reducing the antitumor effector function. Tannic acid, a specific inhibitor of HMGB2, synergized with PD-1 antibody to attenuate tumor growth and reverse T cell exhaustion. Our findings highlight the unique role of HMGB2 as an immune exhaustion associated molecule. Targeting HMGB2 on both CD8$^+$ T cells and tumor cells contributed to promising treatment strategies for HCC.

INTRODUCTION

Primary liver cancer (PLC) is the sixth most common cancer worldwide and the third leading cause of cancer-related death (*1*). Hepatocellular carcinoma (HCC) accounts for ~90% of cases of PLC (*2*). Although patient survival can be improved by curative resection in early and intermediate stages, more than half of patients with HCC are in a locally advanced stage or with distant metastasis at diagnosis. In recent years, breakthroughs have been made in the treatment strategies for advanced liver cancer, with immune checkpoint inhibitors (ICIs) including nivolumab, pembrolizumab, and atezolizumab showing great prospects (*3*). However, because of tumor heterogeneity and immune evasion, drug resistance remains a thorny challenge, preventing patients from sufficient and continuous response to immunotherapy (*4*, *5*).

CD8$^+$ T cells are frontline defense cells against cancer. An inflamed transcriptional signature such as interferon-γ (IFN-γ) response genes is associated with better prognosis and response to anticancer immunotherapies (*5*–*7*). The reduced infiltration and effector function of CD8$^+$ T cells lead to adaptive immune dysfunction and immunotherapy resistance (*8*). Therefore, T cell exhaustion, which is characterized by low secretion of cytotoxic molecules (granzymes and perforin) or effector cytokines (IFN-γ) and high expression of multiple inhibitory receptors (such as PD-1, LAG3, CTLA4, and TIM3), is a critical factor for short duration of ICI treatment response (*9*). Cancer genetic variation, inhibitory tumor immune microenvironment (TIME), metabolic reprogramming, and drug-related damage are leading causes for T cell exhaustion (*10*). Although many anticancer drugs effectively suppress tumor growth by inhibiting key signaling pathways required for cell proliferation, part of them impairs immune cell survival or function. Consequently, developing drugs that target tumor cells and simultaneously enhance, rather than impair, immune function is of great significance.

High-mobility group box 2 (HMGB2) is a highly conserved DNA binding protein that can modify chromatin structure and regulate gene transcription by binding with transcription factors (*11*, *12*), involved in processes such as aging, lipid metabolism, and immune inflammatory response. HMGB2 is highly expressed in multiple solid tumors and is an independent risk factor for poor prognosis of HCC (*13*), but its mechanism for promoting tumor progression has not been thoroughly investigated. A recent study has reported that cell-intrinsic HMGB2 expression is essential for long-term maintenance of exhausted CD8$^+$ T cells during chronic viral infections (*14*). However, the role of HMGB2 in HCC TIME remains unknown.

To identify the previously unknown T cell exhaustion associated molecule, we analyzed human and murine single-cell RNA sequencing (scRNA-seq) data in this study and identified the underlying mechanisms of HMGB2 promoting HCC progression through dual action on effector T cells (T_{EFF}) and cancer cells, respectively. Mechanistically, HMGB2 caused metabolic reprogramming of CD8$^+$ T cells, damaged mitochondrial oxidative phosphorylation (OXPHOS), and dampened the cytotoxicity. Regarding tumor cells, HMGB2 facilitated the transcriptional inhibition of *Stat1* by TRIM24, thereby inactivating the interferon response and reducing the susceptibility and recruitment to effector cells. Last, the specific HMGB2 inhibitor tannic acid effectively synergized with the anti–PD-1 therapy. This study provides a distinctive insight for the immunotherapeutic strategies in HCC treatment.

[1]Department of Liver Surgery, Liver Cancer Institute, Zhongshan Hospital, Fudan University, Key Laboratory of Carcinogenesis and Cancer Invasion of Ministry of Education, Shanghai, China. [2]Research Unit of Liver Cancer Recurrence and Metastasis, Chinese Academy of Medical Sciences, Beijing, China. [3]Department of Thyroid and Breast Surgery, Zhongshan Hospital, Fudan University, Shanghai, China. [4]Department of General Surgery, Zhongshan Hospital, Fudan University, Shanghai, China.
*Corresponding author. Email: shi.yinghong@zs-hospital.sh.cn (Y.-H.S.); tang.zheng@zs-hospital.sh.cn (Z.T.); liu.weiren@zs-hospital.sh.cn (W.-R.L.)
†These authors contributed equally to this work.

RESULTS

Single-cell transcriptomic analysis finds HMGB2 as a previously unknown T cell exhaustion–associated gene

To investigate cellular diversity of T cells, we performed scRNA-seq profiles from seven treatment-naïve HCC samples and four adjacent normal liver tissues. After stringent quality control (QC) filtering, a total of 94,928 cells were assigned to seven cell types (fig. S1, A and B): natural killer (NK)/T cell, myeloid cell, endothelial cell, neutrophil, hepatocyte, fibroblast, and B cell. Top 10 gene markers were listed (table S1). To further address the intrinsic cell heterogeneity, we identified CD8, CD4, and NK populations based on unsupervised algorithm (fig. S1C).

Further clustering of $CD4^+$ T cells yielded five subclusters (fig. S1D), including naïve (CD4-CCR7), T helper (CD4-JUN), effector (CD4-GNLY), effector memory (CD4-GZMK), and regulatory (CD4-CTLA4) T cells. CD4-CTLA4 predominated in the tumor region and CD4-JUN was mostly mapped in the normal liver tissue (fig. S1E). Exhaustion markers including *CTLA4*, *FOXP3*, and *TIGIT* were specifically expressed in regulatory T cells and were significantly elevated in tumors (fig. S1, F and G).

NK cells were categorized into seven clusters (fig. S1H). On the basis of the expression levels of *NCAM1* and *FCGR3A* markers, two major groups were subdivided (*15*). Basically, NK-XCL1, NK-CCL3, and NK-CD160 subclusters were mapped in $CD56^{bright}CD16^{low}$ group, exhibiting a substantial gain of *CD160* expression and secreting chemoattractant genes (*XCL1*, *XCL2*, and *CCL3*). The $CD56^{dim}$ $CD16^{hi}$ group including NK-GZMB, NK-GZMH, NK-GNLY, and NK-KLRC3 exhibited high expression of granzymes and cytotoxic effector genes (fig. S1, I and J).

On the basis of gene expression signature and enriched pathways, we deciphered the functional divergence among $CD8^+$ T cell populations (Fig. 1A and fig. S2A). The first tumor-reactive subcluster, resident memory T cells (T_{RM}), was characterized by specific expression of *NR4A2*, *NR4A3*, and *TUBA4A*, undertaking immune surveillance and immune activation. The second cluster, T_{EFF}, expressing high levels of cytotoxic markers or chemokines *IFNG*, *GZMK*, and *CCL5*, was highly enriched in leukocyte-mediated cytotoxicity and cell killing. The third cluster, naïve T cells (T_N), showed high levels of *CCR7*, *TCF7*, and *LEF1*, functioning as cell differentiation. The fourth cluster, effective memory T cells (T_{EM}), expressed the chemokine receptor CXCR4 and the proinflammatory transmembrane protein *CRTAM*. The fifth cluster, mucosal-associated invariant T cell (MAIT), expressing *SLC4A10* and *KLRB1*, was a highly conserved innate-like T cell subtype exhibiting cell adhesion and differentiation related function (*16*). The remaining $CD8^+$ T cells fell into the sixth cluster, exhausted T cells (T_{EX}), which shared T cell exhaustion markers including *CTLA4*, *PDCD1*, and *TIGIT* (Fig. 1, B and C).

To identify the critical gene mediating T cell exhaustion, we conducted differential analysis between T_{EX} and T_{EFF} subclusters (Fig. 1D). *CTLA4*, *TOX2*, and *HAVCR2* were significantly up-regulated in the T_{EX} group. The same analysis was applied to another two scRNA-seq public datasets (GSE140228 and GSE149614) (*17*, *18*). Subsequently, up-regulated genes in the T_{EX} group were intersected and integrated with a collection of differential genes based on the bulk RNA-seq from a previous study (*19*). *HMGB2* level was consistently increased across four datasets (Fig. 1E), which was highly enriched in the T_{EX} subcluster and poorly expressed in the MAIT, T_{EFF}, and T_{RM} subclusters (Fig. 1F). Cell distribution uniform manifold approximation and projection (UMAP) revealed an aggregation of T_{RM}, T_N, and T_{EFF} in the normal liver tissue (fig. S2B). Tumor-specific $CD8^+$ T cells were characterized by an enrichment of T_{EX} subcluster, with elevated expression of *HMGB2* (fig. S2C). Correlation analysis revealed a negative relationship between *HMGB2* and *IFNG* expression in both tumor and normal liver tissues (fig. S2, D and E). Tumor-specific other than peritumoral *HMGB2* expression was positively correlated with *TIGIT* expression (fig. S2, F and G). This implies that cell-intrinsic HMGB2 possibly hampers $CD8^+$ T cells cytotoxicity. In GSE149614 dataset (Fig. 1G), *HMGB2* expression was relevant with advanced tumor staging (Fig. 1H) and was enriched in the tumor region (Fig. 1I). Similarly, *HMGB2* expression was up-regulated in the tumor region from GSE140228 dataset (Fig. 1, J to L). To allow deeper T cell characterization, we extended our validation to a murine HCC model (*20*). Seven tumors from HCC spontaneous mice were performed scRNA-seq (*20*). $CD8^+$ T cells were clustered into T_{RM}, T_N, T_{EFF}, and T_{EX} (Fig. 1M). *Hmgb2* was highly expressed in T_{EX} and sparsely expressed in T_{EFF}, similar to human HCC (Fig. 1, N and O).

To address the underlying evolution of cellular status among $CD8^+$ T cells, we derived the pseudotime cell trajectory of $CD8^+$ T cells in human and murine tumors using Monocle 2 algorithm (fig. S2, H to M). The differentiation trajectories of human and mouse $CD8^+$ T cells had a similar trend, in which a trajectory started with T_N, then segregated into T_{RM} or T_{EFF}, and lastly ended in T_{EX} or T_{RM} (fig. S2, H and K). The expression of *Hmgb2* and inhibitory immune checkpoint genes gradually rose during the middle and terminal states (fig. S2M). These data suggested that $HMGB2^+$ $CD8^+$ T cell was an important subset in TIME, which probably induced T cell exhaustion and promoted HCC progression.

Hmgb2 deficiency enhances OXPHOS and effector function in $CD8^+$ T cells

To investigate how HMGB2 affects T cell function, we crossed $Hmgb2^{flox/flox}$ mice with *Cd4*-Cre mice to obtain murine models in which *Hmgb2* was conditional knockout (cKO) in $CD8^+$ T cells (*21*) (fig. S3, A and B). No significant difference was observed in spleen sizes and $CD8^+$ T cell apoptosis between *Hmgb2*-cKO and the negative control (NC) mice (fig. S3, C to E). We conducted RNA-seq analysis of isolated NC and cKO $CD8^+$ T cells. The functions of mitochondrial respiratory chain complex assembly and regulation of inflammatory response (Fig. 2A), as well as the pathway of OXPHOS, were enriched in the cKO group, compared to the NC group (Fig. 2B). Most genes involved in the electron transport chain complexes I, II, IV, and V had increased mRNA expression in the cKO group (Fig. 2C). To assess the metabolic consequences of *Hmgb2* deficiency, we applied targeted energy metabolism mass spectrometry (MS) (Fig. 2D). The protein levels of adenosine 5′-triphosphate (ATP), nicotinamide adenine dinucleotide (oxidized form) (NAD^+), and fumaric acid were higher in the activated *Hmgb2*-deficient $CD8^+$ T cells than in NC counterparts (Fig. 2E). Kyoto Encyclopedia of Genes and Genomes (KEGG) metabolic pathway analysis revealed that OXPHOS and mitochondrial electron transport chain were enriched in cKO cells (Fig. 2F). We next assessed $CD8^+$ T cell metabolic fitness. *Hmgb2*-deficient $CD8^+$ T cells displayed increased oxygen consumption rate (OCR) at ATP production, basal respiration, and maximal respiration (Fig. 2, G and H), while the capacities of glycolysis were not affected by *Hmgb2* deficiency (fig. S3, F and G).

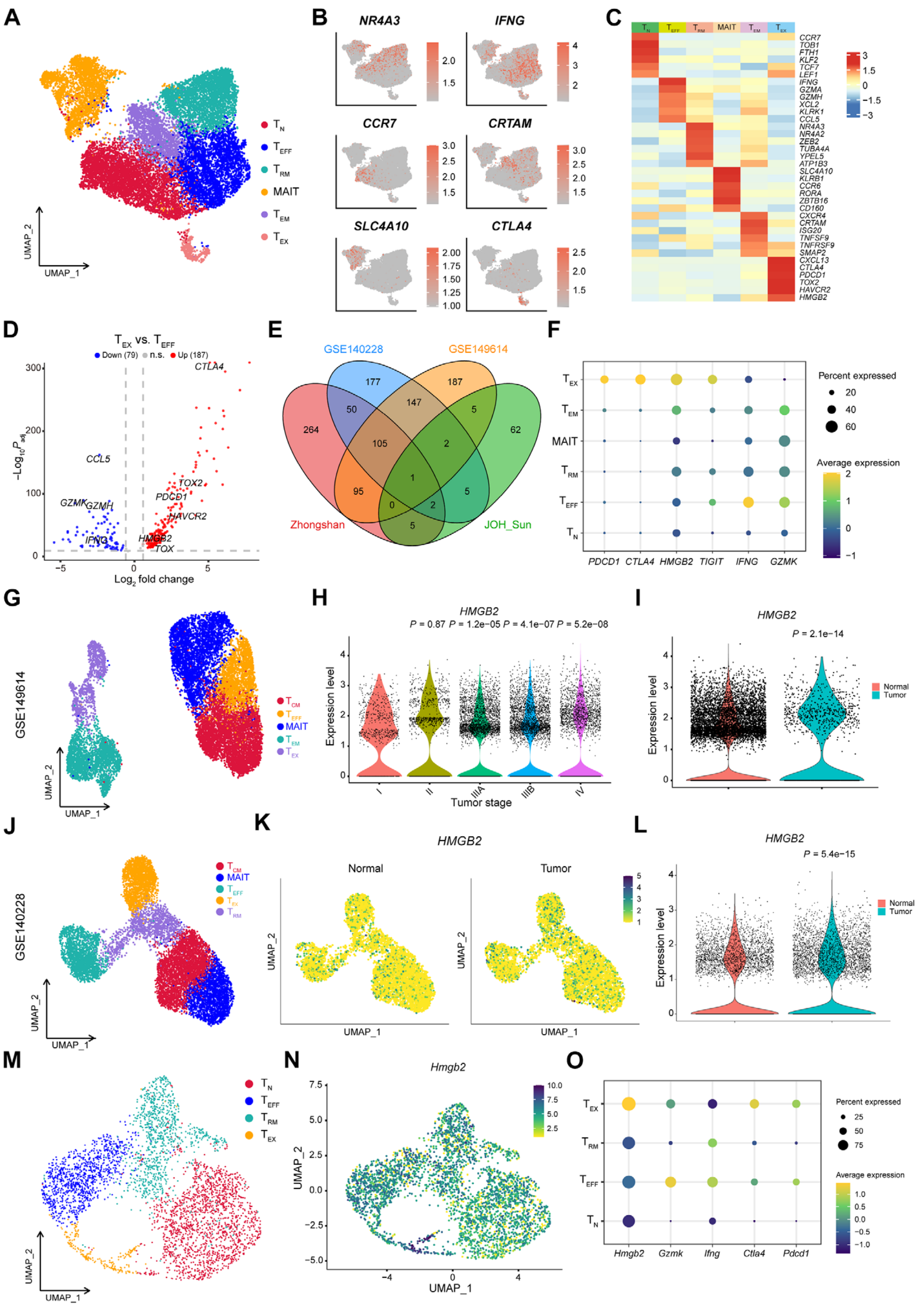

Fig. 1. Identification of HMGB2 as a novel negative regulator for T cell immunity. (**A**) UMAP plot for the different subclusters of CD8$^+$ T cells in human HCC tissues. (**B**) UMAP plots show marker genes in different CD8$^+$ T clusters. (**C**) Heatmap shows cell markers in each CD8$^+$ T subtype. (**D**) Volcano plot for differential expression analysis of T_{EX} and T_{EFF} clusters. P_{adj}, adjusted *P* value; n.s., not significant. (**E**) Venn plot for differential genes between T_{EX} and T_{EFF} clusters in three scRNA-seq datasets and one bulk RNA-seq dataset. (**F**) Dot plot shows specific function markers enriched in different CD8$^+$ T subclusters in Zhongshan cohort. (**G**) UMAP plot for the different subclusters of CD8$^+$ T cells in GSE149614 dataset. T_{CM}, central memory T cells. (**H**) Relative expression of HMGB2 in CD8$^+$ T cells at different tumor stages. (**I**) Relative expression of HMGB2 in CD8$^+$ T cells in the normal liver tissues and tumor tissues. (**J**) UMAP plot for the different subclusters of CD8$^+$ T cells in GSE140228 dataset. (**K**) UMAP plot shows the expression of HMGB2 in different CD8$^+$ T cell subclusters. (**L**) Relative expression of HMGB2 in CD8$^+$ T cells in the normal liver tissues and tumor tissues. (**M**) UMAP plot for the different subclusters of CD8$^+$ T cells in mouse HCC tissues. (**N**) UMAP plot shows *Hmgb2* expression of CD8$^+$ T cells in mouse HCC tissues. (**O**) Dot plot shows specific function markers enriched in CD8$^+$ T subclusters of mouse HCC tissues. Wilcoxon test.

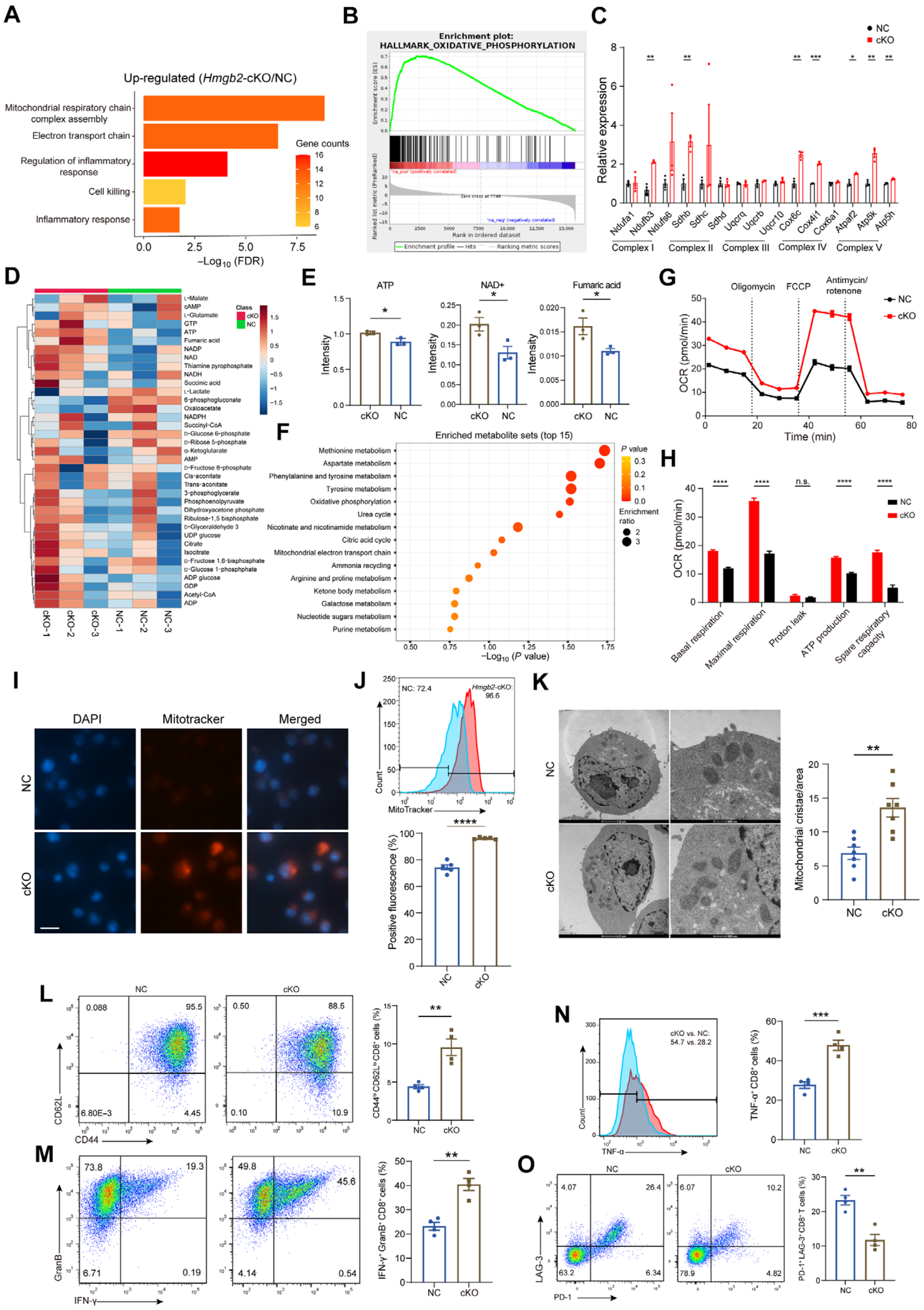

Fig. 2. *Hmgb2* deficiency enhances mitochondrial OXPHOS in CD8⁺ T cells. (**A**) Gene Ontology (GO) analysis reveals changes in *Hmgb2*-cKO CD8$^+$ T cells. FDR, false discovery rate. (**B**) Gene set enrichment analysis (GSEA) shows top pathway enriched in *Hmgb2*-cKO CD8$^+$ T cells. (**C**) mRNA levels of electron transport chain genes in NC and *Hmgb2*-cKO CD8$^+$ T cells. (**D**) Heatmap for energy metabolites in isolated NC and *Hmgb2*-cKO CD8$^+$ T cells detected by liquid chromatography–MS (LC-MS) analysis. cAMP, adenosine 3′,5′-monophosphate; GTP, guanosine 5′-triphosphate; NAD, nicotinamide adenine dinucleotide; NADH, reduced form of NAD$^+$; NADPH, reduced form of nicotinamide adenine dinucleotide phosphate; CoA, coenzyme A; AMP, adenosine 5′-monophosphate; UDP, uridine 5′-diphosphate; ADP, adenosine 5′-diphosphate; GDP, guanosine diphosphate; NADP, beta-nicotinamide adenine dinucleotide phosphoric acid. (**E**) Intensity of ATP, NAD$^+$, and fumaric acid as in (D) ($n = 3$). (**F**) KEGG analysis shows top metabolic pathway changes as in (D). (**G**) Seahorse extracellular flux analysis of OCR in isolated NC and *Hmgb2*-cKO CD8$^+$ T cells. FCCP, carbonyl cyanide *p*-trifluoromethoxyphenylhydrazone. (**H**) Quantification of OCR as in (G) ($n = 32$). (**I**) Immunofluorescence micrographs of NC and *Hmgb2*-cKO OT-I CD8$^+$ T cells stained with MitoTracker (red) and 4′,6-diamidino-2-phenylindole (DAPI) (blue) after coculture with Hepa1-6–OVA cells. Scale bar, 10 μm. (**J**) Comparison of fluorescence of stained MitoTracker as in (I) ($n = 5$). (**K**) Transmission electron microscope images of mitochondria in activated NC and *Hmgb2*-cKO OT-I CD8$^+$ T cells after coculture with Hepa1-6–OVA cells. The density of mitochondrial cristae is compared ($n = 7$). (**L**) Flow cytometry analysis of CD44^{hi} CD62L^{lo} effector CD8$^+$ T cells in NC and *Hmgb2*-cKO OT-I CD8$^+$ T cells after coculture with Hepa1-6–OVA cells ($n = 4$). (**M**) Flow cytometry analysis of GranB$^+$ IFN-γ$^+$ CD8$^+$ T cells as in (L). (**N**) Flow cytometry analysis of TNF-α$^+$ CD8$^+$ T cells as in (L). (**O**) Flow cytometry analysis of PD1$^+$ LAG-3$^+$ CD8$^+$ T cells as in (L). Data are presented as the means ± SEM. *$P < 0.05$; **$P < 0.01$; ***$P < 0.001$; ****$P < 0.0001$. Student's *t* test for (C), (E), and (J) to (O). Two-way analysis of variance (ANOVA) test for (H).

After crossing *Hmgb2*$^{flox/flox}$;*Cd4*-Cre mice with OT-I mice, we isolated NC and *Hmgb2*-cKO OT-I CD8$^+$ T cells. To evaluate the influence of HMGB2 on effector function, we conducted flow cytometry and electron microscopy analysis on NC and *Hmgb2*-cKO OT-I CD8$^+$ cells with or without tumor stimulation by Hepa1-6–ovalbumin (OVA) cells. *Hmgb2* deficiency in CD8$^+$ T cells led to a boost in active mitochondrial content (Fig. 2, I and J), filamentous and obvious mitochondria, and tight cristae structures (Fig. 2K) in both tumor stimulation and bystander status (fig. S4, A to C). The proportion of CD44^{hi} CD62^{lo} effector CD8$^+$ T cells was much higher in *Hmgb2*-deficient CD8$^+$ T cells than in NC counterparts (Fig. 2L and fig. S4D). Specifically, the infiltration of GranB$^+$ IFN-γ$^+$ CD8$^+$ T cells and tumor necrosis factor–α (TNF-α)$^+$ CD8$^+$ T cells were elevated in the cKO group (Fig. 2, M and N, and fig. S4, E and F), while the exhausted PD-1$^+$ LAG-3$^+$ CD8$^+$ T cell cluster was reduced (Fig. 2O). However, the exhausted subsets barely differed in nonantigen stimulation status (fig. S4, G and H), suggesting that HMGB2-induced T cell exhaustion phenotypes might rely on constant tumor antigen stimulation. Together, these data illustrated that *Hmgb2* deficiency in CD8$^+$ T cells contributed to mitochondrial OXPHOS intensification and enhanced cytotoxic immunity.

HMGB2 regulated mitochondrial transcription through KEAP1/NRF2 signal

Given the importance of HMGB2 in chromatin remodeling, we performed assay for targeting accessible-chromatin with high-throughout sequencing (ATAC-seq) to explore notable changes in chromatin accessibility in the absence of HMGB2. A higher frequency of chromatin accessibility at the promoter regions of *Hmgb2*-deficient T cells was detected (Fig. 3A). Subsequently, we focused on the mitochondrial transcription factors and immunotoxicity-related genes. Increased accessibility near the promoter of *Tfam*, *Tfb1m*, *Ifng*, and *Gzmb* was observed in the *Hmgb2*-cKO cells (Fig. 3B), suggesting a potential positive correlation between mitochondrial transcriptional activity and cytotoxicity. On the basis of mouse scRNA-seq data, negative correlations between the expression levels of *Hmgb2*, *Tfam*, and *Tfb1m* were observed (Fig. 3C), which was confirmed by quantitative polymerase chain reaction (PCR) and western blotting experiments (fig. S4, I and J).

It is reported that NRF2, predominantly modulated by KEAP1 via ubiquitination, activates TFAM and TFB1M, thereby enhancing the transcriptional activity of mitochondrial electron transport chains (*22*–*25*). Therefore, we hypothesized that HMGB2 affected mitochondrial function by intervening this signaling axis. The protein expression rather than mRNA level of NRF2 increased in the *Hmgb2*-deficient CD8$^+$ T cells (Fig. 3D and fig. S4K). In addition, the transcription of antioxidant response element (ARE) genes was activated by *Hmgb2* knockout (Fig. 3E). These results suggested that HMGB2 down-regulated NRF2 expression at the protein level. To uncover the potential mechanisms of HMGB2-mediated ubiquitination on NRF2, we conducted coimmunoprecipitation (CoIP) assay. HMGB2 led to a rising ubiquitination of NRF2 (Fig. 3, F and G). Adding recombinant HMGB2 protein in vitro inhibited NRF2 and TFAM protein levels and triggered NRF2 ubiquitination, which could be abolished by KEAP1–NRF2–IN-1 (IN-1), a binding inhibitor of KEAP1 and NRF2 (Fig. 3, H and I).

To validate this in vivo, we performed multicomplex immunofluorescence (mIF) assay in HCC spontaneous tumor tissues. cKO tumors had higher infiltration of NRF2$^+$ KEAP1$^-$ CD8$^+$ T cells and lower infiltration of KEAP1$^+$ CD8$^+$ T cells (Fig. 3J).

The influence of the HMGB2/KEAP1/NRF2 axis on mitochondrial oxidative respiratory function was then evaluated. Recombinant HMGB2 protein reduced the OXPHOS level of NC CD8$^+$ T cells, and this downward trend was reversed by IN-1 (Fig. 3, K and L), suggesting that the impact of HMGB2 on mitochondrial function depended on the KEAP1/NRF2 axis. Together, these results indicated that HMGB2 up-regulated the expression of KEAP1, promoting NRF2 ubiquitination and degradation through KEAP1-NRF2 interaction.

Hmgb2 depletion in T cells synergized with PD-1 antibody

scRNA-seq for HCC lesions scheduled to neoadjuvant anti–PD-1 monotherapy was performed to evaluate the role of HMGB2$^+$ CD8$^+$ T cells in ICI efficacy from our previous study (*26*). Among the four cases, two were diagnosed as partial pathological response (PR), and the others were diagnosed as nonresponse. Five CD8$^+$ T cell types were identified (Fig. 4A). The nonresponse group had a higher proportion of T_{EX} and a lower proportion of T_{EFF} (Fig. 4B). The expression of *HMGB2* in the CD8$^+$ T cells was up-regulated in the nonresponse subgroup (Fig. 4, C and D) and was significantly increased after PD-1 antibody treatment in the nonresponse group (Fig. 4E). Furthermore, we validated the findings using a public dataset based on the neoadjuvant immunotherapy for colorectal cancer (*27*). A total of 12,098 CD8$^+$ T cells were assigned to MAIT, intraepithelial lymphocyte (IEL), T_{EM}, T_{EX}, and one tissue-resident memory–mitotic subset (fig. S5A). The composition of cells was highly heterogeneous between pathological complete response (pCR) and non-pCR subgroups (fig. S5B). Non-pCR patients had a larger population of HMGB2$^+$ CD8$^+$ T cells compared to pCR patients (fig. S5C). Together, these results indicated that decreasing HMGB2$^+$ CD8$^+$ T cells predicted effective ICI treatment.

To explore the effect of *Hmgb2* in antitumor T cell response, we challenged NC and *Hmgb2*-cKO mice with Hepa1-6 HCC cells. Compared to NC group, cKO mice displayed a markedly decreased tumor burden with prolonged survival (fig. S5, D to F). Both CD8$^+$ and IFN-γ were mostly infiltrated in the cKO group receiving PD-1 antibody (fig. S5, G to I).

To better understand the influence of HMGB2 on HCC occurrence and development, we applied hydrodynamic tail vein injection spontaneous model (Fig. 4F). The combination of *Hmgb2*-cKO mice and PD-1 antibody maximally inhibited the tumor growth and prolonged the survival time (Fig. 4, G to I). In addition, treating cKO mice with PD-1 antibody increased the frequency of IFN-γ$^+$ CD8$^+$ T cells and GranB$^+$ CD8$^+$ T cells compared to NC counterparts (Fig. 4, J to L). Immunofluorescence demonstrated that ICI-treated *Hmgb2*-cKO mice induced an increase in effector marker expression, suggesting an immunostimulatory status (Fig. 4M).

To discern whether *Hmgb2*-deficient CD4$^+$ T cells contributed to HCC progression, we validated the pathway changes and antitumor effect in vitro and in vivo. *Hmgb2* depletion in CD4$^+$ T cell did not boost NRF2 expression and downstream gene transcription (fig. S6, A to C). Next, we cocultured NC or *Hmgb2*-cKO CD4$^+$ T cells with Hepa1-6–OVA cells and OT-I CD8$^+$ cells to detect the modulation on CD8$^+$ T cell activity. No significance was observed between two groups regarding the proportion of IFN-γ$^+$ TNF-α$^+$ CD8$^+$ T cells (fig. S6, D and E). T cell depletion assay showed that blocking CD8 attenuated the tumor suppression effect brought by *Hmgb2*$^{flox/flox}$;*Cd4*-Cre mice, while tumor growth was not affected by CD4 antibody. Similar phenomena were also observed in *Hmgb2*-cKO

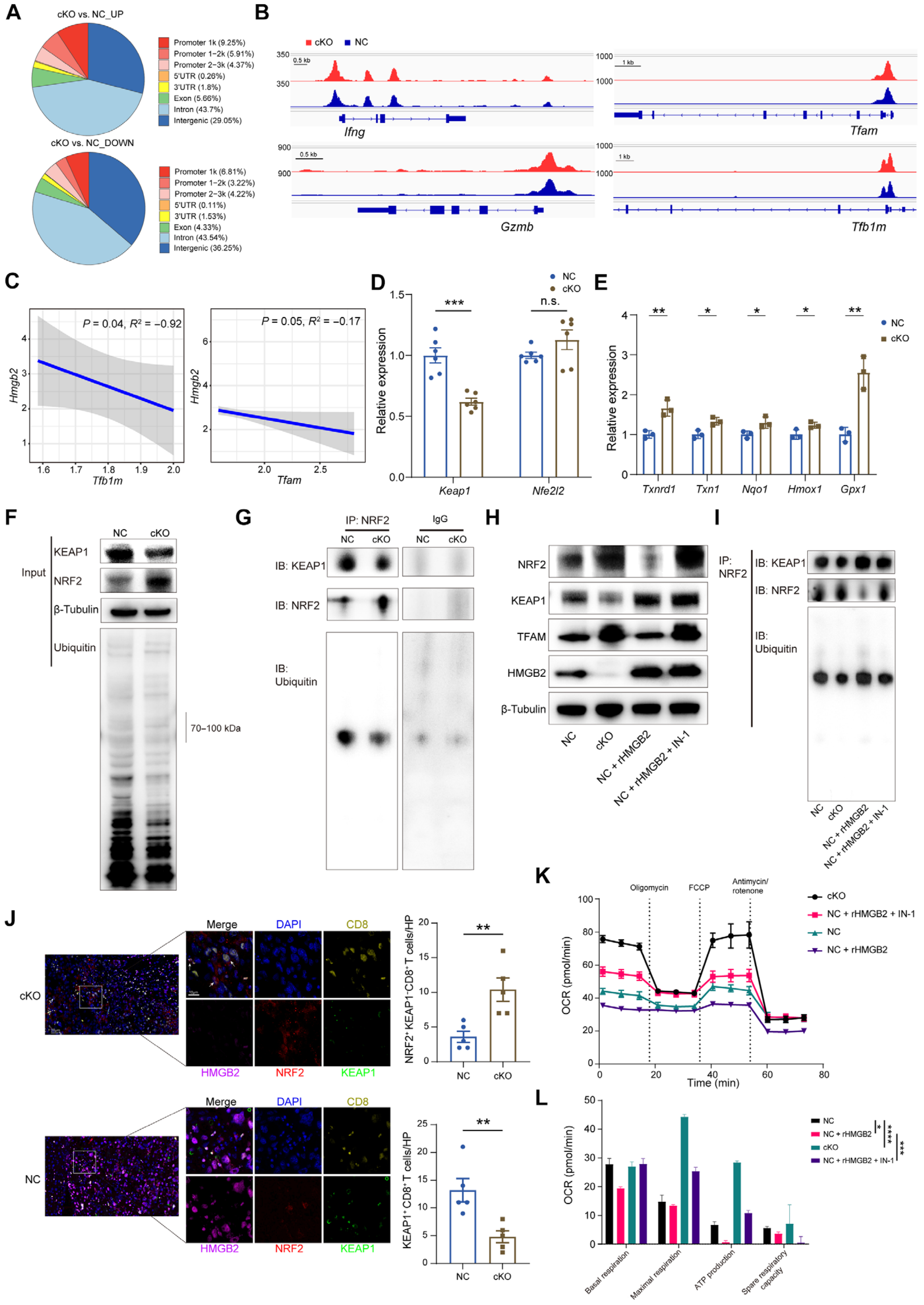

Fig. 3. HMGB2 regulated mitochondrial transcription through KEAP1/NRF2 pathway. (**A**) Location of differential accessible ATAC-seq peaks in NC and *Hmgb2*-cKO CD8$^+$ T cells. 3′UTR, 3′ untranslated region; 5′UTR, 5′ untranslated region. (**B**) Chromatin accessibility changes of genes associated with effector function and mitochondrial transcription factors. (**C**) The correlation between *Hmgb2*, *Tfam*, and *Tfb1m* expression in CD8$^+$ T cells from murine scRNA-seq data. (**D**) mRNA levels of *Keap1* and *Nfe2l2* in NC and *Hmgb2*-cKO CD8$^+$ T cells ($n = 6$). (**E**) mRNA levels of ARE genes in NC and *Hmgb2*-cKO CD8$^+$ T cells ($n = 3$). (**F**) Protein changes of KEAP1 and NRF2 after *Hmgb2* knockout. (**G**) The ubiquitination of NRF2 after *Hmgb2* knockout. IB, immunoblot. (**H**) Protein changes of KEAP1 and NRF2 after stimulation of mouse recombinant HMGB2 protein and IN-1. NC CD8$^+$ T cells were treated with recombinant HMGB2 protein (500 ng/ml) and/or IN-1 (10 μM) for 48 hours. (**I**) The ubiquitination of NRF2 after cell treatment as in (H). (**J**) Immunofluorescence staining of spontaneous HCC tissues in NC and *Hmgb2*-cKO mice. NRF2$^+$ KEAP1$^-$ CD8$^+$ T cells were labeled. Scale bars, 20 μm (left) and 10 μm (right). HP, high power field. (**K**) Seahorse extracellular flux analysis of OCR in different CD8$^+$ T cell groups ($n = 6$ to 8) after cell treatment as in (H). (**L**) Quantification of seahorse extracellular flux analysis of OCR of different CD8$^+$ T cells as in (K). Data are presented as the means ± SEM. *$P < 0.05$; **$P < 0.01$; ***$P < 0.001$; ****$P < 0.0001$. Pearson test for (C) Student's *t* test for (D), (E), and (J). Two-way ANOVA test for (L).

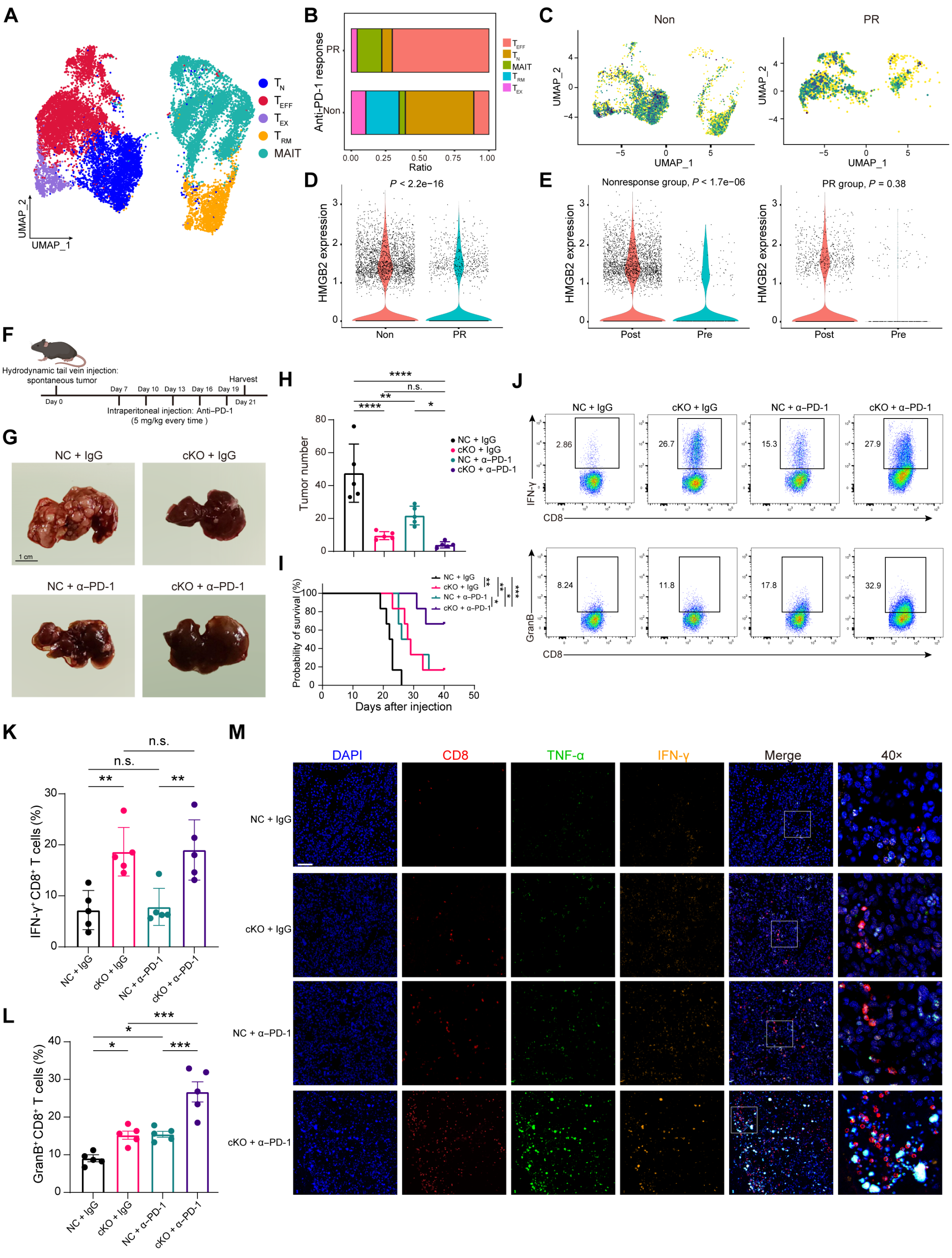

Fig. 4. HMGB2$^+$ CD8$^+$ T cells dampens antitumor immunity and immunotherapeutic response. (**A**) UMAP plot for the different subclusters of CD8$^+$ T cells in human HCC tissues scheduled to neoadjuvant anti–PD-1 monotherapy. (**B**) Histogram plot shows the proportions of CD8$^+$ T cells as in (A). (**C**) UMAP plot for the HMGB2 expression of CD8$^+$ T cells in nonresponse and PR HCC tissues. (**D**) Relative expression of HMGB2 in CD8$^+$ T cells as in (C). (**E**) Comparison of HMGB2 expression in CD8$^+$ T cells in pretreatment and postoperative tissues. (**F**) The schematic diagram shows the medication regimen in vivo. (**G**) Representative images of HCC spontaneous models in different treatment groups. Scale bar, 1 cm. (**H**) Tumor numbers of HCC spontaneous models as in (G). (**I**) Survival time of HCC spontaneous models as in (G). (**J**) Flow cytometry of intratumoral IFN-γ$^+$ CD8$^+$ T cells and GranB$^+$ CD8$^+$ T cells from spontaneous HCC tissues. (**K**) Quantification of intratumoral IFN-γ$^+$ CD8$^+$ T cells ($n = 5$). (**L**) Quantification of intratumoral GranB$^+$ CD8$^+$ T cells ($n = 5$). (**M**) Immunofluorescence staining of effector markers in spontaneous HCC tissues as in (G). Scale bar, 50 μm. Data are presented as the means ± SEM. *$P < 0.05$; **$P < 0.01$; ***$P < 0.001$; ****$P < 0.0001$. Wilcoxon test for (D) and (E). One-way ANOVA test for (H), (K), and (L). The icon in (F) is cited from BioRender: W. Qu (2025; https://BioRender.com/m63n001).

mice receiving anti–PD-1 treatment (fig. S6, F and G). Overall, HMGB2$^+$ CD8$^+$ T cell was predictive of poor HCC immunotherapy response and *Hmgb2*$^{flox/flox}$;*Cd4*-Cre mice improved the efficacy of anti–PD-1 therapy.

HMGB2 knockdown in tumor cells enhanced IFN-γ response

HMGB2 has been reported to be a classic oncogene in various solid tumors based on bulk or scRNA transcriptome sequencing (fig. S7, A and B) (*13*). HMGB2 expression in HCC was significantly higher than that in the normal liver tissue (fig. S7, C and D). Patients with high expression of HMGB2 had poor overall survival and recurrence-free survival (fig. S7, E and F). The Cancer Genome Atlas-Liver Hepatocellular Carcinoma (TCGA-LIHC) dataset validated the carcinogenic role of HMGB2 (fig. S7, G and H). To examine the influence of HMGB2 on proliferation, we disrupted HMGB2 in human and murine HCC cell lines by short hairpin RNA (shRNA) interference. Knockdown of HMGB2 in the Huh7 and Hepa1-6 cell lines suppressed cell proliferation in vitro (fig. S8, A to E).

To determine the antitumor effect of HMGB2 in vivo, we challenged BALB/C nude mice and immunocompetent C57BL/6J mice with Hepa1-6 shCtrl and shHmgb2 cells, respectively. Knocking down *Hmgb2* attenuated the growth of subcutaneous tumors in both C57BL/6J mice and nude mice (Fig. 5, A to C). To further assess the role of the TIME in antitumor effect, we compared the differences in tumor volumes between shCtrl and shHmgb2 group in two types of mice. The difference in C57BL/6J mice was higher than the counterparts in nude mice (Fig. 5D). These data demonstrated that knocking down *Hmgb2* within tumor cells inhibited HCC growth, partially due to the impact of tumor microenvironment (TME).

To uncover the molecular mechanisms of HMGB2 promoting HCC progression, we performed RNA-seq and ATAC-seq on Hepa1-6 cell lines. shHmgb2 cells showed strong activation of the response to IFN-γ and regulation of T cell activation (Fig. 5, E and F). Signal transducers and activators of transcription 1 (STAT1) is a key signal transducer of IFN-γ pathway that favors innate and adaptive immune responses (*28*, *29*). Increased accessibility of *Stat1* promotor was observed in the shHmgb2 cells (fig. S9A). Accordingly, protein levels of STAT1, phosphorylated STAT1 (p-STAT1), and fatty acid synthase (FAS) were markedly increased by HMGB2 knockdown, while the pathway changes were restored by STAT1 inhibitor fludarabine (Fig. 5G and fig. S9B).

To quantitatively evaluate the IFN-γ response, we detected the apoptosis by exogenous stimulation of IFN-γ. More early-apoptotic cells were detected in the knockdown group than the control counterparts (Fig. 5H). In addition, with the IFN-γ concentration increasing from 10 to 20 ng/ml, the proportion of elevated early-apoptotic cells was significantly higher in the knockdown group (Fig. 5I). T cell killing assay further demonstrated that *Hmgb2* knockdown augmented susceptibility to T cell–mediated cytotoxicity (Fig. 5J).

Next, we conducted in vitro assays to characterize the downstream transcripts of STAT1. *Hmgb2* knockdown increased the mRNA levels of interferon-stimulated genes (ISGs) and cytotoxic chemokines represented by *Cxcl10* and *Ccl5* (fig. S9C). RNA-seq showed that *CXCL10* was up-regulated in both human and mouse knockdown cell lines (fig. S9D), consistent with the enzyme-linked immunosorbent assay (ELISA) results (fig. S9, E and F). Thus, we speculated that inhibiting HMGB2 contributed to an inflammatory tumor environment by promoting CXCL10 secretion. Terminal deoxynucleotidyl transferase–mediated deoxyuridine triphosphate nick end labeling (TUNEL) staining showed an increase in apoptotic cells in the shHmgb2 subcutaneous tumors (Fig. 5K). In addition, the number of CXCL10-, CD8-, and IFN-γ–positive staining increased in the knockdown group (Fig. 5, L and M). Collectively, *Hmgb2* knockdown bolstered STAT1-mediated IFN-γ response and promoted CXCL10 secretion, with recruitment of effector CD8$^+$ T cells into HCC TIME.

Hmgb2 knockdown improved immunotherapy efficacy through TRIM24/STAT1 axis

As HMGB2 bound to DNA without sequence specificity to increase the chromatin accessibility to transcription factors (*30*), we hypothesized that HMGB2-induced *STAT1* transcriptional inhibition depended on the intensified binding of transcription repressors to *STAT1*. The CUT&Tag sequencing detected a binding peak around the transcription start site (TSS) of *Stat1* promoter (fig. S9G). Chromatin immunoprecipitation (ChIP) assay confirmed the direct interaction between HMGB2 and *Stat1* chromatin (Fig. 6A). These results indicated that HMGB2 bound to the *Stat1* promoter and possibly acted as a cofactor in transcriptional regulation.

Previous study has reported that TRIM24 acts on the retinoic acid–responsive element (RARE) in the *Stat1* promoter region, silencing Stat1 transcription (*31*). In TCGA-LIHC dataset, *TRIM24* expression was associated with poor prognosis of patients with HCC (fig. S9H). Knocking down *Trim24* activated the expression of STAT1 and p-STAT1 (fig. S9I) and promoted cell apoptosis (fig. S9, J and K). These data revealed that TRIM24 had a negative regulatory effect on STAT1.

We next focused on the influence of HMGB2 on TRIM24/STAT1 axis. The mIF assay of HCC subcutaneous tumors depicted the colocalization among HMGB2, TRIM24, and STAT1 (Fig. 6B). The direct interaction between HMGB2 and TRIM24 was validated by CoIP assay (Fig. 6C). Subsequently, ChIP assay revealed that TRIM24 bound with RARE region at the promoter of *Stat1* and was regulated by HMGB2 (Fig. 6D). Further, a luciferase reporter assay demonstrated that dual overexpression of *Hmgb2* and *Trim24* exhibited a stronger effect than single-gene intervention, while small interfering RNA (siRNA) targeting Trim24 (siTrim24) reversed the inhibitory effect by overexpression of *Hmgb2* (Fig. 6E). These findings indicated that HMGB2 enhanced the binding between TRIM24 and *Stat1* chromatin, thus inhibiting transcription of *Stat1*.

To elaborate the role of STAT1 in vivo, we transfected shHmgb2 Hepa1-6 cells with *Stat1* overexpression lentivirus and challenge C57BL/6J mice with subcutaneous tumors (fig. S10, A and B). A lessened trend of tumor volume was observed by *Stat1* overexpression (fig. S10C). Previous studies have reported that CXCL10/CXCR3 signaling is required for T cell tumor infiltration and tumor immunotherapy (*32*). Since *Hmgb2* knockout did not up-regulate CXCR3 expression in CD8$^+$ T cells at transcriptome and protein level (fig. S10, D to F), we inferred that enhancement of T cell chemotactic activity depended merely on chemokines released by tumor cells after *HMGB2* silencing. To examine whether the antitumor effect was mediated through CXCL10/CXCR3 signaling, we performed CXCR3 inhibitor (AMG487) treatment assay. The infiltration of IFN-γ$^+$ TNF-α$^+$ CD8$^+$ T cells was partially diminished by AMG487 after coculture with shHmgb2-OVA cells in vitro (fig. S10, G and H). Moreover, the regression of tumor growth by *Hmgb2* disruption was partially abrogated by CXCR3 inhibitor treatment (fig. S10, I and J). Together, the findings revealed that

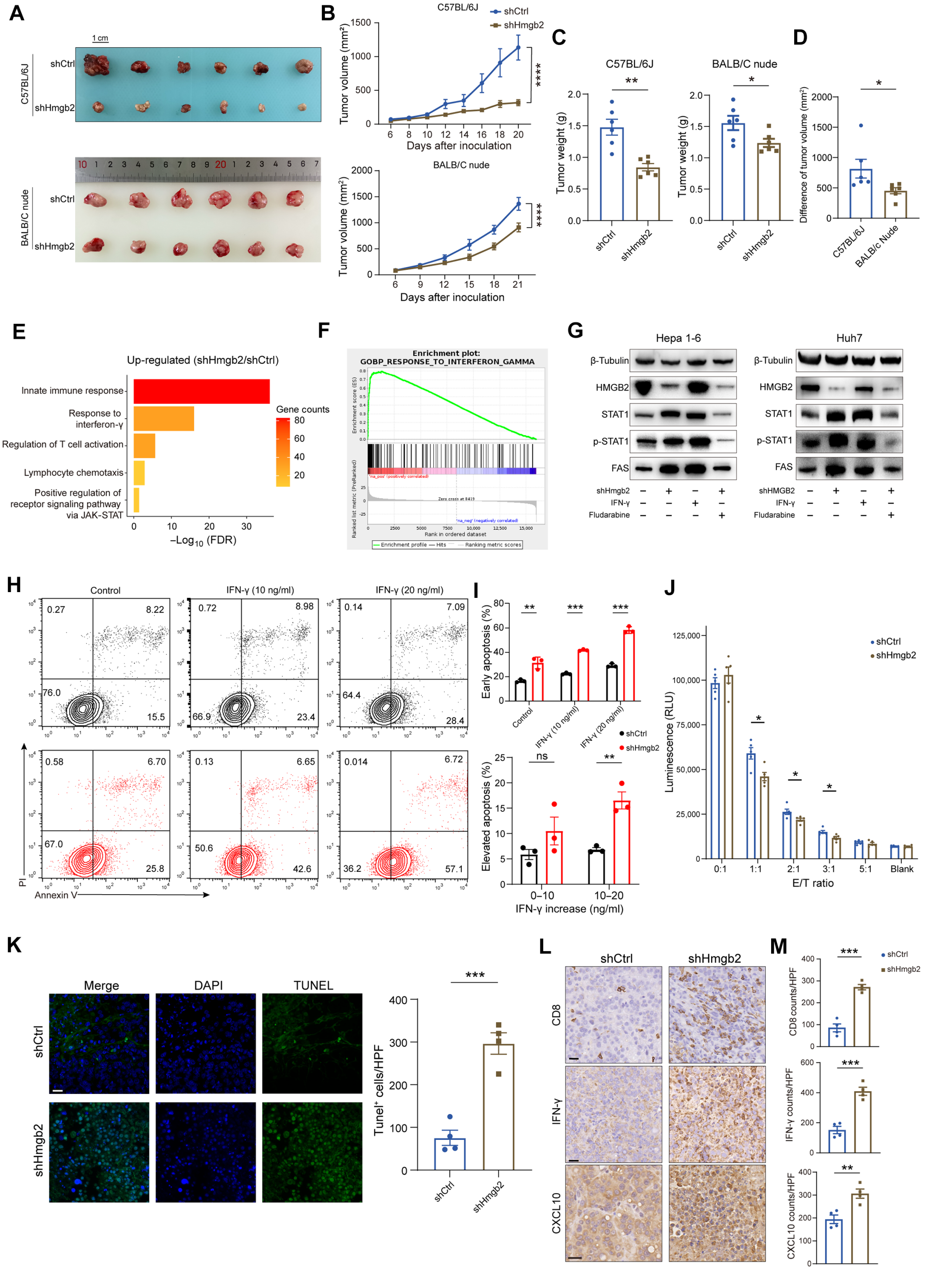

Fig. 5. *Hmgb2* knockdown enhanced IFN-γ response in HCC cells. (**A**) Representative images of harvested Hepa1-6 subcutaneous HCC tumors. Scale bar, 1 cm (**B**) Tumor growth curves of Hepa1-6 subcutaneous tumors in BALB/C nude mice and C57BL/6J mice. (**C**) Tumor weights of subcutaneous Hepa1-6 tumors in BALB/C nude mice and C57BL/6J mice ($n = 6$). (**D**) Difference of tumor volumes between shCtrl and shHmgb2 subcutaneous tumors in BALB/C nude mice and C57BL/6J mice ($n = 6$). (**E**) Differential GO pathways in Hepa1-6 shHmgb2 cells. JAK, Janus kinase. (**F**) GSEA analysis shows top pathway enriched in Hepa1-6 shHmgb2 cells. (**G**) Western blotting experiment shows STAT1 pathway changes in Hepa1-6 cells and Huh7 cells. Cells were treated with IFN-γ (10 ng/ml) or fludarabine (10 μM) for 24 hours. (**H**) Annexin V apoptosis analysis for Hepa1-6 cells treated with vehicle and IFN-γ (10 or 20 ng/ml). PI, propidium iodide. (**I**) Quantification for proportions of apoptotic cells after treatment of IFN-γ. (**J**) T cell killing assay with Hepa1-6 shCtrl and shHmgb2 cells and wild-type (WT) $CD8^+$ T cells ($n = 5$). RLU, relative light unit. (**K**) Terminal deoxynucleotidyl transferase–mediated deoxyuridine triphosphate nick end labeling (TUNEL) staining and quantification of Hepa1-6 subcutaneous tumors ($n = 4$). Scale bar, 25 μm. HPF, high power field. (**L**) Representative immunohistochemistry images of CD8, IFN-γ, and CXCL10 staining in Hepa1-6 subcutaneous tumors. Scale bars, 20 μm. (**M**) Quantification of CD8, IFN-γ, and CXCL10 staining in Hepa1-6 subcutaneous tumors ($n = 4$). Data are presented as the means ± SEM. $*P < 0.05$; $**P < 0.01$; $***P < 0.001$. Two-way ANOVA test for (B) Student's t test for (C), (D), (I), (J), (K), and (M).

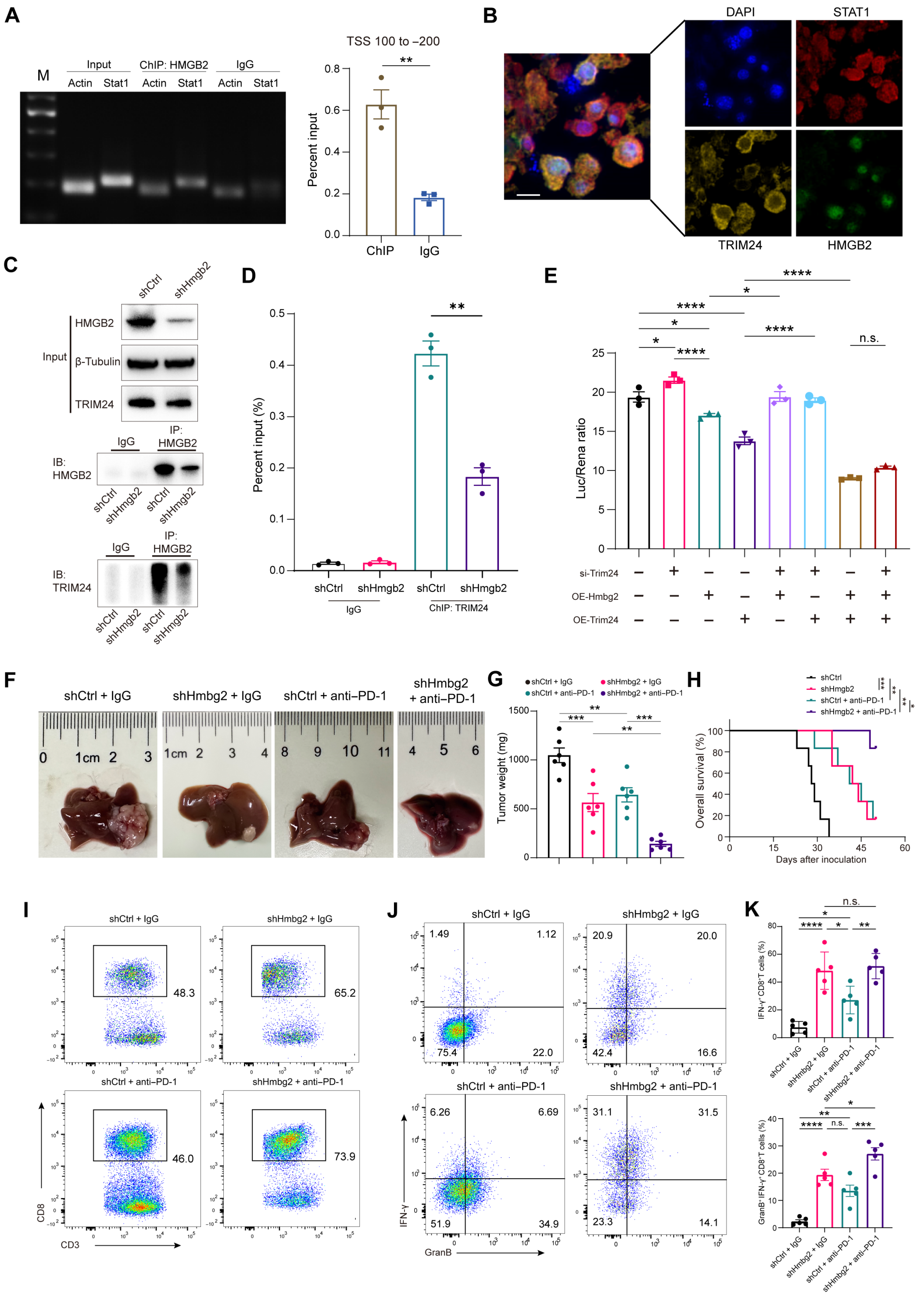

Fig. 6. HMGB2 impaired antitumor immunity through Trim24/Stat1 signal. (**A**) ChIP assay shows the interaction of HMGB2 with *Stat1* chromatin ($n = 3$). (**B**) Colocalization of HMGB2, STAT1 and TRIM 24 in HCC subcutaneous tumor tissue. Scale bar, 10 μm. (**C**) CoIP assay shows the interaction of HMGB2 and TRIM24. (**D**) ChIP-PCR shows that TRIM24 modulates the transcriptional level of *Stat1* ($n = 3$). (**E**) Luciferase reporter assay shows that the *Trim24/Hmgb2* signal modulates the transcriptional level of *Stat1* ($n = 3$). OE, overexpression. (**F**) Representative images of the orthotopic HCC models. (**G**) Tumor weights of different groups from the orthotopic HCC models ($n = 6$). (**H**) Overall survival of different treatment groups from the orthotopic HCC models. (**I**) Flow cytometry of intratumoral $CD3^+$ $CD8^+$ T cells as in (G). (**J**) Flow cytometry of intratumoral effector markers as in (G). (**K**) Quantification of intratumoral IFN-γ^+ $CD8^+$ T cells and $GranB^+$ $CD8^+$ T cells in different treatment groups ($n = 5$). Data are presented as the means ± SEM. *$P < 0.05$; **$P < 0.01$; ***$P < 0.001$; ****$P < 0.0001$. Student's *t* test for (A) and (D). One-way ANOVA test for (E), (G), and (K).

Hmgb2 deficiency–induced tumor regression depended on STAT1/CXCL10/CXCR3 pathway.

Then, using an orthotopic HCC model in immunocompetent mice, we demonstrated that *Hmgb2* knockdown in tumor cells combined with PD-1 antibody effectively reduced tumor burden (Fig. 6, F and G) and extended survival time (Fig. 6H). Moreover, *Hmgb2* knockdown increased the infiltration of $CD8^+$ T cells (Fig. 6I) and boosted IFN-γ^+ $CD8^+$ T cells in tumors (Fig. 6, J and K). These data indicated that *Hmgb2* knockdown in tumor cells impeded tumor growth and altered the immunosuppressive microenvironment.

Targeting *Hmgb2* synergizes with immunotherapy

Since *Hmgb2* knockout within $CD8^+$ T cells promoted IFN-γ secretion and *Hmgb2* knockdown within tumor cells enhanced IFN-γ response, we reasoned that blocking HMGB2 resulted in positive feedback killing cycle between T cells and tumor cells. NC and *Hmgb2*-cKO OT-I $CD8^+$ T cells were directly or indirectly cocultured with Hepa1-6 shCtrl and shHmgb2 cells transfected with OVA, respectively (fig. S11A). T cell killing assay revealed that dual intervention on two cell types optimized the killing effect of $CD8^+$ T cells (fig. S11, B to D). Flow cytometry analysis was performed to evaluate the coculture influence on T cells. Dual blockade of *Hmgb2* expression in tumor cells and T cells significantly stimulated TNF-α and IFN-γ secretion of $CD8^+$ T cells (fig. S11, E to H). These findings suggested that cointervention of *Hmgb2* expression within $CD8^+$ T cells and tumor cells synergistically improved IFN-γ–mediated killing capacity.

To screen the specific inhibitor of HMGB2, we conducted Cell Counting Kit-8 (CCK-8) toxicity assay and surface plasmon resonance (SPR) assay using a natural product library containing 3720 compounds. Drugs with low cytotoxicity were eliminated. Tannic acid (T0801) ranked the top regarding the affinity with murine HMGB2 protein, with a response unit value around 40 (fig. S12A). SPR assay on different concentrations of tannic acid (fig. S12B) revealed a "slow binding and slow dissociation" pattern. The molecular docking experiment depicted the amino acid binding mode (fig. S12C).

In vitro, the median inhibitory concentration (IC_{50}) value of tannic acid was 21.23 μM (Fig. 7A). Exogenous stimulation of tannic acid boosted the expression of p-STAT1 in Hepa1-6 cells (fig. S12D) and the mRNA levels of ISGs (fig. S12E). In $CD8^+$ T cells, the OXPHOS level was significantly elevated (fig. S12F), and the cytotoxic markers including GranB, TNF-α and IFN-γ were up-regulated by tannic acid (fig. S12, G and H). To confirm the specificity of tannic acid in vivo, we performed tannic acid treatment in *Hmgb2*-disrupted tumors. Intake of tannic acid caused tumor regression compared to shHmgb2 group, which probably attributed to the effect on $CD8^+$ T cells. In contrast, no significant difference was reached in tumor volumes between *Hmgb2*-cKO mice inoculated with shHmgb2 Hepa1-6 cells receiving tannic acid or not (fig. S12, I and J). Together, tannic acid served as the specificity inhibitor for HMGB2 in vivo, which failed to control tumor growth after *Hmgb2* disruption in tumor cell and $CD8^+$ T cell. These findings supported that tannic acid inhibited the downstream activities of HMGB2.

To investigate the antitumor effect of tannic acid combined with PD-1 antibody, we established murine HCC orthotopic model and subcutaneous tumor model of colorectal cancer. The combination therapy exhibited marked antitumor efficacy in two cancer types (Fig. 7, B to E). $CD8^+$ T cells were increased after tannic acid medication regardless of ICI treatment in colorectal cancer (Fig. 7F). To further determine the efficacy of combination therapy in HCC development, we treated spontaneous HCC tumors with single agents (tannic acid or anti–PD-1 therapy) and the combination regimen. The results showed that the combination therapy significantly reduced the tumor burden and prolonged the survival (Fig. 7, G to I). In addition, the combination therapy altered the immune landscape toward antitumor immunity, characterized by increased proportions of $CD8^+$ T cells, especially IFN-γ^+ and $GranB^+$ subsets, and decreased proportions of T_{EX} with high expression of TIM-3, PD-1, and LAG-3 (Fig. 7, J and K). These results fully demonstrated that tannic acid enhanced the efficacy of PD-1 antibody, increased the infiltration of effector $CD8^+$ T cells, and reversed T cell exhaustion.

DISCUSSION

T cell exhaustion is a formidable challenge to antitumor immunotherapy. In this study, we used scRNA-seq on human and mouse HCC tumor tissues to thoroughly characterize the diversity of intratumoral $CD8^+$ T cells and identified T cell exhaustion–associated genes. $HMGB2^+$ $CD8^+$ T cells were originally identified as a proimmune suppression subset in HCC TME that induced anti–PD-1 treatment resistance. The study unveiled the underlying mechanisms that HMGB2 boosts tumor growth by dual action on T cells and cancer cells. The combination regimen potentiated a more effective treatment strategy.

Given the importance of scRNA-seq in investigating TME including T cells (*27*, *33*), we jointly analyzed three scRNA-seq datasets and one bulk RNA-seq dataset, covering different etiologies causing HCC, to excavate the potential therapeutic targets regarding T_{EX}. Collectively, HMGB2 was highly expressed in the T_{EX} and positively correlated with advanced tumor stage. These findings originally demonstrated the relationship between $HMGB2^+$ $CD8^+$ T cells and cancer malignancy, emphasizing the crucial function of HMGB2 in HCC TME. Through in vitro and in vivo assays, we proved that HMGB2 enhanced KEAP1-mediated ubiquitination degradation of NRF2, suppressed the expression levels of mitochondrial transcription factors TFAM and TFB1M, and impaired oxidative respiratory function by disrupting the transcription of downstream electron transport chains. The mechanisms shed light on the distinctive role of HMGB2 in mitochondrial energy metabolism and clarify the process by which OXPHOS damage leads to $CD8^+$ T cell dysfunction.

Understanding the metabolic reprogramming of T cells may improve the strategies aimed at "reinvigorating" T_{EX} subset (*34*, *35*). During differentiation, naïve $CD8^+$ T cells primarily use oxidative degradation metabolism to maintain the fundamental ATP supply (*36*, *37*). Under antigen stimulation, the overall metabolic profile of T_{EFF} shifts toward aerobic glycolysis to acquire more energy for killing function (*38*). Memory T cells, on the other hand, rely more on fatty acid oxidation and OXPHOS to meet their energy requirements (*39*). The role of OXPHOS in effector $CD8^+$ T cells remains controversial. It has been reported that $CD8^+$ T cells with high OXPHOS signature can predict immune treatment resistance; however, the multiomics analysis has not been experimentally tested (*40*). Other studies propose that transient enhancement of OXPHOS maintains T cell metabolic adaptability and boosts effector function (*41*, *42*). In this study, *Hmgb2* deficiency increases the level of OXPHOS and exerts a stronger killing effect but does not affect glycolytic metabolism. These imply that HMGB2 regulates the regional mitochondrial function rather than the general metabolic differentiation of T cells.

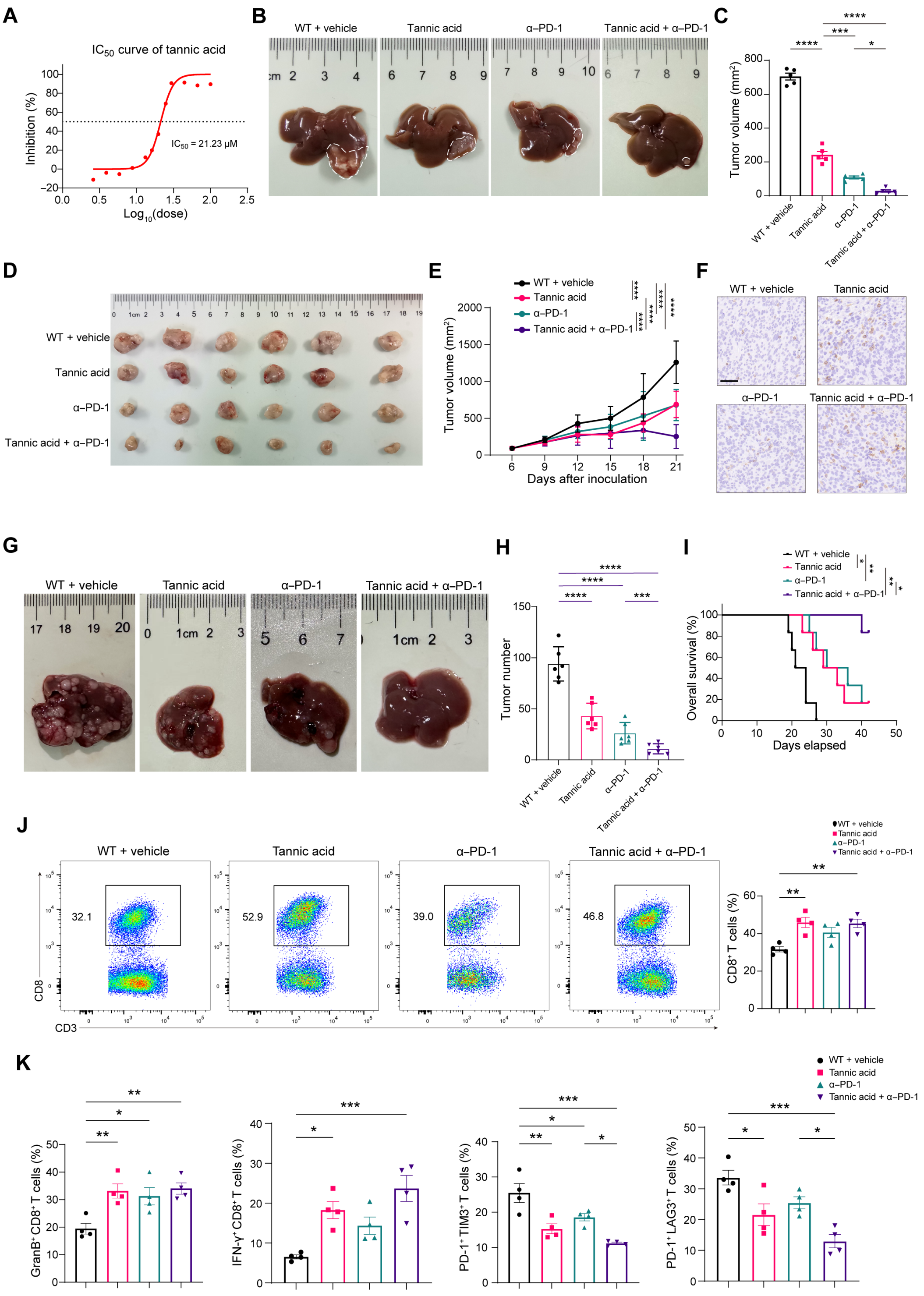

Fig. 7. Tannic acid synergizes with anti–PD-1 immunotherapy. (**A**) Proliferative inhibition curve for tannic acid on Hepa1-6 cells. $IC_{50} = 21.23$ μM. (**B**) Representative images of orthotopic HCC models. (**C**) Tumor volumes of different groups from the orthotopic HCC model ($n = 6$). (**D**) Representative images of colorectal subcutaneous tumors constructed by MC38 cell injection ($n = 6$). (**E**) Tumor growth curves of subcutaneous tumors constructed by MC38 cell injection ($n = 6$). (**F**) Immunohistochemistry staining images of CD8 in the subcutaneous tumors constructed by MC38 cell injection. Scale bar, 50 μm. (**G**) Representative images of spontaneous HCC model. (**H**) Tumor weights of different treatment groups in the spontaneous HCC model ($n = 6$). (**I**) Overall survival of different treatment groups in the spontaneous HCC model ($n = 6$). (**J**) Flow cytometry and quantification of intratumoral $CD3^+$ $CD8^+$ T cells as in (H). (**K**) Quantification of flow cytometry analysis on intratumoral effector $CD8^+$ T cells and exhausted $CD8^+$ T cells as in (H). Data are presented as the means ± SEM. *$P < 0.05$; **$P < 0.01$; ***$P < 0.001$; ****$P < 0.0001$. One-way ANOVA test for (C), (H), (J), and (K). Two-way ANOVA test for (E).

Our study also reveals an important role of HMGB2$^+$ CD8$^+$ T cells in immunotherapy. High expression of HMGB2 in CD8$^+$ T cells indicated treatment resistance to immunotherapy in both HCC and colorectal cancers. In vivo experiments further confirmed that *Hmgb2*$^{flox/flox}$;*Cd4*-Cre mice combined with PD-1 antibody maximally attenuated tumor progression and promoted the infiltration of T_{EFF} with killing function. This coincided with our unveiled in vitro mechanisms. According to a recent study, HMGB2$^-$ CD8$^+$ T cells promoted malignancy, although HMGB2 maintained the exhausted status of CD8$^+$ T cells in melanoma and chronic viral infections (*14*). The discrepancy was possibly caused by the heterogeneity of different cancer types and mouse models. Furthermore, no sequencing data or in vivo experiments related to immunotherapy were indicated in this study. In summary, *Hmgb2*-deficient CD8$^+$ T cells reverse T cell exhaustion and reduce tumor burden in HCC.

The exact process by which HMGB2 accelerates the growth of HCC cells is yet unclear. The present study originally reported the role of HMGB2 in the innate immune system. HMGB2 knockdown upregulated p-STAT1 and activated the IFN-γ response pathway. On one hand, this amplified cell apoptosis due to increasing expression of FAS. On the other hand, chemokine CXCL10 transcribed by STAT1 recruited more CD8$^+$ T cells, especially T_{EFF}, into the tumor local immunological milieu. In vivo, HMGB2 promoted HCC growth on two levels, as demonstrated by the subcutaneous tumor differences between immunocompetent and immunodeficient mouse. The differences of tumor volumes between shHmgb2 group and shCtrl group were more significant in the presence of immune function. This section provides insights that HMGB2 influences tumor TME in addition to tumor proliferation.

We found that shHmgb2 HCC cells were more susceptible to CD8$^+$ T cells or exogenous IFN-γ. In the CD8$^+$ T cell counterpart, *Hmgb2* knockout release higher cytokine IFN-γ and cytotoxin GranB. T cell killing assay illustrated that dual intervention on HMGB2 stimulated a positive killing loop. These findings further support that HMGB2 inhibitor tannic acid primes an efficient antitumor immunity by inducing intratumoral IFN-γ boost. Inhibiting HMGB2 in two cell types may "turn cold tumors hot," diminishing the immunosuppressive immune microenvironment (*43*). For patients receiving anti–PD-1 therapy across various cancer indications, a robust IFN-γ signature has been found to be an efficacious predictor of treatment response (*4*, *7*, *44*). Here, HMGB2 blockade represents a promising strategy to increase the curative efficiency of the anti–PD-1 therapy in both HCC and colorectal cancer, attributed to augmented IFN-γ$^+$ CD8$^+$ T cell infiltration. The limitation of this study lies in the applicability of tannic acid, which is initially designed on the basis of murine protein binding assay. Validation on human tumor tissues merits further studies.

In conclusion, our study finds a previously unknown T cell exhaustion–associated target at the single-cell level and reveals mechanisms by which HMGB2 impairs mitochondrial OXPHOS in CD8$^+$ T cells and inhibits IFN-γ response in tumor cells, thus inducing immune evasion of HCC. Inhibiting HMGB2 effectively synergizes with PD-1 antibody, potentiating compelling targets for immunotherapy.

MATERIALS AND METHODS

Human and murine specimens

Seven HCC tumor tissues and four paired adjacent normal liver tissues were collected at Zhongshan Hospital of Fudan University for scRNA-seq, as reported in a previous study (*20*). scRNA-seq data based on seven tumors from spontaneous HCC mouse models were used for validation (*20*). Then, four patients from Zhongshan Hospital scheduled to neoadjuvant anti–PD-1 therapy were enrolled for scRNA-seq including pretreatment biopsy tumor tissues and postoperative tumor tissues. Treatment response was evaluated according to the mRECIST (*45*). Tissue microarrays contained 233 HCC and peritumoral normal liver samples were used for immunohistochemistry assay (*46*). All patients were informed about the collection of their clinical information and resected tissues before surgery, with a signature of consent form. The study obtained ethical approval from the Institutional Review Board of Zhongshan Hospital (B2021-611) and complied with the standards of the Declaration of Helsinki.

Single-cell RNA sequencing

Fresh tumor and adjacent liver tissue samples were cut into ~1-mm^3 pieces in the Dulbecco's modified Eagle's medium (DMEM) (BIOAGRIO) and were dissociated and digested according to the previous study (*18*). Cells were loaded onto the 10x Chromium Single Cell Platform (10x Genomics) for a target recovery of 10,000 single cells (Single Cell 3′ library and Gel Bead Kit v.3.1) as described in the manufacturer's protocol. Generation of gel beads in emulsion (GEMs), barcoding, GEM reverse transcription, complementary DNA amplification, and library construction were all performed according to the manufacturer's instructions. Qubit was used for library quantification before pooling. The final library pool was sequenced on the Illumina NovaSeq 6000 instrument using 150–base pair paired-end reads. GEM creation, library construction, sequencing, and CellRanger analysis were performed by Shanghai Xu Ran Biotechnology Co. Ltd. (www.xurangene.com).

T cell killing assay

CellTiter-Lumi II Luminescent Cell Viability Assay Kit (C0056M, Beyotime) and Crystal Violet Staining Solution (C0121, Beyotime) were used to study the live tumor cells after direct coculture with tumor cells and CD8$^+$ T cells. Hepa1-6 shCtrl and shHmgb2-OVA cells were washed with phosphate-buffered saline (PBS), and 10,000 tumor cells were added per well in flat-bottom 96-well plates or 120,000 tumor cells per well in flat-bottom 24-well plates. OT-I CD8 T cells were added at different effector-to-target (E/T) ratios. Following 24 hours of coculture, the media were removed, and wells were washed with PBS to remove dead tumor cells and CD8$^+$ T cells. Live, adherent tumor cells were then counted using the CellTiter-Lumi II Luminescent Cell Viability Assay Kit (96-well plates) or fixed and stained with crystal violet (24-well plates).

T cell isolation and stimulation

Naïve CD8$^+$ T cells and CD4$^+$ T cells were sorted using the EasySep Mouse T Cell Isolation Kit (19853 and 19852, STEMCELL Technologies). The sorted cells were stimulated with mouse recombinant interleukin-2 (IL-2; 50 ng/ml), anti-CD3 (5 μg/ml), and anti-CD28 (5 μg/ml) antibodies in six-well plates (1×10^6 cells per well) for 48 to 72 hours. Flow cytometry analysis, immunoblot analysis, transmission electron microscope, and metabolic analysis were performed after stimulation.

Cell culture

Huh7 human HCC cells, Hepa1-6 murine HCC cells, and MC38 murine colon cancer cells were purchased from cell bank of Chinese

Academy of Science (Shanghai) and cultured in DMEM supplemented with 10% fetal bovine serum (FBS) and penicillin-streptomycin (100 U/ml). Isolated $CD8^+$ T cells were cultured in RPMI 1640 supplemented with 10% FBS, penicillin-streptomycin (100 U/ml), and stimulants.

Hepa1-6–OVA cells were collected and inoculated in the bottom chamber of the six-well plate at a density of 4×10^5 cells/2.5 ml 24 hours before coculture. After cell adherence, activated OT-I $CD8^+$ T cells were collected and suspended in complete medium and plated in the top chamber (six-well insert; pore size, 0.4 μm; Pullen, China) at a density of 1×10^6 cells/1.6 ml. After coculture for 24 to 48 hours, further assays were performed on collected OT-I $CD8^+$ T cells.

scRNA-seq data processing and cell clustering

For QC of scRNA-seq data, we set the cutoff min.cells as 3 and min. features as 300 by Seurat package (V4.3.0). Then, cells with high expression of mitochondrial genes and hemoglobin related genes were filtered out. The Harmony algorithm (*47*) was used to integrate cells from different tumor samples. After normalization and data scale, we used RunPCA to perform dimension reduction and FindNeighbors and FindClusters to cluster cells. Cell types were named according to the marker genes reported in previous literature and were visualized using UMAP dimensionality reduction.

Differential gene expression

The FindAllMarkers function was used to perform differential gene expression analysis of different cellular populations and set adjusted $P = 0.05$ with log fold change > 0.25 and pct.1 > 0.3 as the cutoff value.

Cell trajectory inference

To explore the differentiation trajectory of intratumor T cells, we performed inferencing algorithm Monocle 2 (V2.28.0) (*48*) to infer the pseudotime of each cell. ggpubr (V0.6.0) and ggplot2 (V3.4.3) packages were used to visualize the trajectories of different cell types and targeted genes.

High-throughput CUT&Tag sequencing

CUT&Tag assay was performed as described previously with modifications (*49*). Briefly, 100,000 cells were washed and incubated with 10 μl of concanavalin A–coated magnetic beads (Bangs Laboratories) at room temperature (RT) for 10 min. Then, unbound supernatant was removed, and bead-bound cells were resuspended with wash buffer and a 1:50 dilution of primary antibody or immunoglobulin G (IgG) control antibody (normal rabbit IgG, catalog no. 12-370, Millipore; anti-HMGB2, ab67282, Abcam) and incubated on a rotating platform overnight at 4°C. Next, the primary antibody was removed, secondary antibody (anti-rabbit IgG antibody monoclonal, AP132, Millipore) was diluted 1:100 in wash buffer, and cells were incubated at RT for 60 min. A 1:100 dilution of pA-Tn5 adapter complex was prepared in dig-med buffer [0.01% digitonin, 20 mM Hepes (pH 7.5), 300 mM NaCl, 0.5 mM spermidine, and 1× protease inhibitor cocktail] and incubated with cells at RT for 1 hour. Then, cells were resuspended in tagmentation buffer and incubated at 37°C for 1 hour. DNA was purified using phenol–chloroform–isoamyl alcohol extraction and ethanol precipitation. To amplify libraries, 21 μl of DNA was mixed with 2 μl of a universal i5 and a uniquely barcoded i7 primer. A volume of 25 μl of NEBNext High-Fidelity 2× PCR Master Mix was added. The sample was placed in a thermocycler with a heated lid using the following cycling conditions: 72°C for 5 min (gap filling), 98°C for 30 s, 14 cycles of 98°C for 10 s and 63°C for 30s, and final extension at 72°C for 1 min and hold at 8°C. The size distribution of libraries was determined by Agilent 4200 TapeStation analysis, and libraries were mixed to achieve equal representation as desired aiming for a final concentration as recommended by the manufacturer. Sequencing was performed in the Illumina NovaSeq 6000 using 150–base pair paired-end reads following the manufacturer's instructions.

ATAC-seq library preparation and analysis

ATAC-seq was performed according to ATAC-seq protocol by Shanghai Jiayin Biotechnology Ltd. Briefly, cells were harvested from cell culture and lysed in lysis buffer. The Nextera DNA Library Preparation Kit (Illumina) was used to perform the transposition according to the manufacturer's manual. A total of 50,000 nuclei were pelleted, resuspended with transposase, and incubated for 30 min at 37°C. The transposed DNA fragments were purified immediately after with a MinElute PCR Purification Kit (QIAGEN). Samples were PCR-amplified using 1× NEBNext High-Fidelity PCR Master Mix (New England Biolabs, MA). Subsequent libraries were purified with the MinElute PCR Purification Kit (QIAGEN) and subjected to sequencing on Illumina NovaSeq 6000 using PE150.

Raw reads of fastq format were first processed through in-house perl scripts. Clean data (clean reads) were obtained by removing reads containing adapter, reads containing ploy-N, and low-quality reads from raw data. Next, Q20, Q30, and GC content of the clean data were calculated. All the downstream analyses were based on the clean data with high quality. Pair-end reads were then aligned to mouse mm10 reference genome using bwa program (V0.7.17). The bam file was generated by the unique mapped reads as an input file, using MACS2 software (V2.7.1) for callpeak with cutoff $q < 0.05$.

Targeted energy metabolism MS detection

The metabolites were extracted from cell residue (5×10^6 cells) with 1 ml of precooled methanol/acetonitrile/water (v/v, 2:2:1) under sonication for 1 hour in ice baths. The mixture was incubated at −20°C for 1 hour, followed by centrifugation at 14,000*g* for 20 min at 4°C, and then transferred to the sampling vial for liquid chromatography–MS (LC-MS) analysis. To ensure data quality for metabolic profiling, QC samples were prepared by pooling aliquots of all samples that were representative of all samples under analysis and used for data normalization. The LC-MS portion of the platform was based on a Shimadzu Nexera X2 LC-30 AD system equipped with an ACQUITY UPLC BEH amide column (1.7 μm, 2.1 mm by 100 mm; Waters) and a triple quadruple mass spectrometer (QTRAP 5500, AB SCIEX). Metabolites were detected in electrospray negative-ionization and positive-ionization mode. The 2-μl samples were injected sequentially with LC autosampler. The ACQUITY UPLC BEH amide column (1.7 μm, 2.1 mm by 100 mm; Waters) was heated to 45°C under a flow rate of 300 μl/min. A gradient was used to separate the compounds consisted of 20 mM ammonium acetate and 5% acetonitrile with pH 9.45 (solvent A) and 100% acetonitrile (solvent B). The gradient started at 5% solvent A for 1 min, increased linearly to 45% solvent A over 12 min, and then increased linearly to 60% solvent A over 1 min with a 2-min hold before returning to the starting mixture during 0.1 min and reequilibrating for 3 min. QC samples were injected every six or eight samples during acquisition.

The MS conditions were set according to the manufacturer's instructions. To construct the metabolite multiple reaction monitoring (MRM) library, each metabolite standard (50 mg/ml) was first

analyzed by LC-MS/MS to get the optimal MRM transition parameters. Then, the retention time of 40 energy-related metabolites was determined by measuring the corresponding MRM (Q1/Q3) transition individually. A serial dilution of reference standard including 40 energy-related metabolites was prepared for LC-MS analysis.

Raw MRM data files were processed by peak finding, alignment, extraction, and filtering using MultiQuant software. The discriminating metabolites were obtained using a statistically significant threshold of two-tailed Student's *t* test (*P* value) on the normalized raw data. Metabolites with a *P* value less than 0.05 were considered statistically significant metabolites. To identify the perturbed biological pathways, the differential metabolite data were performed KEGG pathway analysis using KEGG database (http://geneontology.org/). KEGG enrichment analyses were carried out with the Fisher's exact test.

Lentivirus and plasmids

To generate the stable knockdown cell lines, lentivirus packaging shRNA plasmids were designed and provided by Genomeditech. The sequence of the effective shRNAs were provided as follows: shHMGB2-1, GCAAAGGAGAAGTCGAAGTTT; shHMGB2-2, GGCCAACAGGCTCAAAGAAGA; shHmgb2-1, TTGGAGATACTGCGAAGAAAC; shHmgb2-2, AGAGCGACAAAGCTCGTTATG. A nontargeting, scrambled silencing RNA was used as control (shCTRL). Virus packaging was performed in 293T cells after cotransfection of packaging plasmids PGMLV-hU6-MCS-CMV-Puro-WPRE. The overexpression lentivirus of Stat1 was packaged in the plasmid of PGMLV-CMV-Mouse_Stat1 × Flag-PGK-Puro. The sequence was provided as follows: GCCACCATGTCACAGTGGTT
CGAGCTTCAGCAGCTGGACTCCAAGTTCCTGGAGCAG-
GTCCACCAGCTGTACGATGACAGTTTCCCCATGGAAAT-
CAGACAGTACCTGGCCCAGTGGCTGGAAAAGCAAGACTGG-
GAGCACGCTGCCTATGATGTCTCGTTTGCGACCATCC-
GCTTCCATGACCTCCTCTCACAGCTGGACGACCAGTA-
CAGCCGCTTTTCTCTGGAGAATAATTTCTTGTTGCAGCA-
CAACATACGGAAAAGCAAGCGTAATCTCCAGGATAACTTC-
CAAGAAGATCCCGTACAGATGTCCATGATCATCTACAACT-
GTCTGAAGGAAGAAAGGAAGATTTTGGAAAATGCCCAAA-
GATTTAATCAGGCCCAGGAGGGAAATATTCAGAACACT-
GTGATGTTAGATAAACAGAAGGAGCTGGACAGTAAAGT-
CAGAAATGTGAAGGATCAAGTCATGTGCATAGAGCAG-
GAAATCAAGACCCTAGAAGAATTACAAGATGAATAT-
GACTTTAAATGCAAAACCTCTCAGAACAGAGAAGGT-
GAAGCCAATGGTGTGGCGAAGAGCGACCAAAAACAG-
GAACAGCTGCTGCTCCACAAGATGTTTTTAATGCTT-
GACAATAAGAGAAAGGAGATAATTCACAAAATCAGAGA-
GTTGCTGAATTCCATCGAGCTCACTCAGAACACTCTGAT-
TAATGACGAGCTCGTGGAGTGGAAGCGAAGGCAG-
CAGAGCGCCTGCATCGGGGGACCGCCCAACGCCTGCCTG-
GATCAGCTGCAAAGCTGGTTCACCATTGTTGCAGAGA-
CCCTGCAGCAGATCCGTCAGCAGCTTAAAAAGCTGGAG-
GAGTTGGAACAGAAATTCACCTATGAGCCCGACCCTATTA-
CAAAAAACAAGCAGGTGTTGTCAGATCGAAC-
CTTCCTCCTCTTCCAGCAGCTCATTCAGAGCTCCTTCGTG-
GTAGAACGACAGCCGTGCATGCCCACTCACCCGCAGAG-
GCCCCTGGTCTTGAAGACTGGGGTACAGTTCACTGT-
CAAGCTGAGACTGTTGGTGAAATTGCAAGAGCTGAA-
CTATAACTTGAAAGTGAAAGTCTCATTTGACAAAGATGT-
GAACGAGAAAAACACAGTTAAAGGATTTCGGAAGTTCAA-
CATCTTGGGTACGCACACAAAAGTGATGAACATGGAA-
GAATCCACCAACGGAAGTCTGGCAGCTGAGTTCCG-
ACACCTGCAACTGAAGGAACAGAAAAACGCTGGGAA-
CAGAACTAATGAGGGGCCTCTCATTGTCACC-
GAAGAACTTCACTCTCTTAGCTTTGAAACCCAGTTGTGC-
CAGCCAGGCTTGGTGATTGACCTGGAGAC-
CACCTCTCTTCCTGTCGTGGTGATCTCCAACGTCAGC-
CAGCTCCCCAGTGGCTGGGCGTCTATCCTGTGGTACAA-
CATGCTGGTGACAGAGCCCAGGAATCTCTCCTTCTTCCT-
GAACCCCCCGTGCGCGTGGTGGTCCCAGCTCTCAGAGGT-
GTTGAGTTGGCAGTTTTCATCAGTCACCAAGAGAGGTCT-
GAACGCAGACCAGCTGAGCATGCTGGGAGAGAAGCT-
GCTGGGCCCTAATGCTGGCCCTGATGGTCTTATTCCATG-
GACAAGGTTTTGTAAGGAAAATATTAATGATA-
AAAATTTCTCCTTCTGGCCTTGGATTGACAC-
CATCCTAGAGCTCATTAAGAAGCACCTGCTGTGCCTCTG-
GAATGATGGGTGCATTATGGGCTTCATCAGCAAGGAGC-
GAGAACGCGCTCTGCTCAAGGACCAGCAGCCAGGGAC-
GTTCCTGCTTAGATTCAGTGAGAGCTCCCGGGAAGGGGC-
CATCACATTCACATGGGTGGAACGGTCCCAGAACGGAG-
GTGAACCTGACTTCCATGCCGTGGAGCCCTACAC-
GAAAAAAGAACTTTCAGCTGTTACTTTCCCAGATAT-
TATTCGCAACTACAAAGTCATGGCTGCCGAGAACATAC-
CAGAGAATCCCCTGAAGTATCTGTACCCCAATATT-
GACAAAGACCACGCCTTTGGGAAGTATTATTCCAGAC-
CAAAGGAAGCACCAGAACCGATGGAGCTTGACGACCCTA-
AGCGAACTGGATACATCAAGACTGAGTTGATTTCTGT-
GTCTGAAGTCCACCCTTCTAGACTTCAGACCACAGA-
CAACCTGCTTCCCATGTCTCCAGAGGAGTTTGATGAGAT-
GTCCCGGATAGTGGGCCCCGAATTTGACAGTATGATGAG-
CACAGTAggatcc. Puromycin (4 μg/ml; Beyotime) was used to kill nontransfected cells.

To generate the transient knockdown cell lines, siRNA plasmids were transfected into cells in logarithmic growth with Lipofectamine 3000 (Thermo Fisher Scientific). The sequences of the effective siRNAs were provided as follows (5′-3′): siTrim24-1, CAUUGAUGGUGGAGAUAAAtt; siTrim24-2, GCAAGCGGCUGAUUACAUAtt.

Enzyme-linked immunosorbent assay

Chemokine concentrations of human and mouse CXCL10 were measured by ELISA kits according to the manufacturer's instructions. The ELISA kits used in this study included human CXCL10 ELISA kit (Ab83700, Abcam) and mouse CXCL10 ELISA kit (LEM717-2, LAIZEE).

Immunoprecipitation and immunoblotting

Cells were washed with PBS and lysed on ice for 30 min in radioimmunoprecipitation assay buffer containing protease inhibitor. Next, cells were ultrasonically lysed for 10 s, three rounds in total, with a power setting of 37%. Cell lysates were immunoprecipitated with the appropriate antibodies using protein A/G agarose beads. Samples were then diluted using SDS loading buffer (Beyotime) and were denatured by being boiled for 10 min. Immunoblotting was conducted following SDS–polyacrylamide gel electrophoresis (ACE Biotechnology), transferring to a polyvinylidene fluoride membrane (Merck Millipore), and incubation with specific antibodies.

Transmission electron microscope

NC and *Hmgb2*-deficient $CD8^+$ T cells were stimulated with anti-CD3 (5 μg/ml) and anti-CD28 (5 μg/ml) in the presence of recombinant

Table 1. Markers and article numbers of antibodies. PE, phycoerythrin; FITC, fluorescein isothiocyanate; APC, antigen-presenting cell; HRP, horseradish peroxidase; mAb, monoclonal antibody.

Markers	No.	Company
Anti-human/mouse HMGB2	T56970	Abmart, China
Anti-human/mouse HMGB2	Ab67282	Abcam, USA
Fixable Viability Dye eFluor 780	65-0865-14	Thermo Fisher Scientific, USA
Anti-mouse CD45 BV510	103138	BioLegend
Anti-mouse CD3 PE-Cy7	100320	BioLegend
Anti-mouse CD8 PerCP-Cy5.5	551162	BD, USA
Anti-mouse NK1.1 PE	108707	BioLegend
Anti-mouse CD4 FITC	557307	BD, USA
Anti-mouse CD25 BV421	102043	BioLegend
Anti-mouse Foxp3 APC	00-5523-00	Thermo Fisher Scientific, USA
Anti-mouse CD366 FITC Tim3	11-5870-82	Thermo Fisher Scientific, USA
Anti-mouse CD223 BV421 Lag3	740072	BD, USA
Anti-mouse CD279 PE PD-1	12-9985-81	Thermo Fisher Scientific, USA
Anti-mouse TNF-α FITC	11-7321-81	Thermo Fisher Scientific, USA
Anti-mouse IFN-γ BV421	505830	BioLegend
Anti-human/mouse GranB PE	372207	BioLegend
Anti-mouse CD44 PE	MA5-17880	Thermo Fisher Scientific, USA
Anti-mouse CD62L APC	17-0621-82	Thermo Fisher Scientific, USA
Anti-mouse CD195 (CCR5) PE	107005	BioLegend
Anti-mouse CD183 (CXCR3) APC	155905	BioLegend
Anti-human/mouse KEAP1	10503-2-AP	Proteintech, China
Anti-human/mouse KEAP1	MB66441	Bioworld, China
Anti-human/mouse TFAM	Ab307302	Abcam, USA
Anti-human/mouse TFB1M	MB65372	Bioworld, China
Anti-human/mouse β-tubulin	M30109	Abmart, China
Goat anti-mouse IgG (H + L) HRP	SB-AB0102	Sharobio, China
Goat anti-rabbit IgG (H + L) HRP	SB-AB0101	Sharobio, China
Anti-human/mouse NRF2	16396-1-AP	Proteintech, China
Anti-mouse IgG	B30010	Abmart, China
Anti-human/mouse ubiquitin	BS62242	Bioworld, China
Anti-human/mouse TNF-α	PY19810	Abmart, China
Anti-human/mouse IFN-γ	BS40422	Bioworld, China
Purified NA/LE hamster anti-mouse CD3e	553057	BD, USA
Purified NA/LE hamster anti-mouse CD28	553294	BD, USA
Anti-human/mouse CD8	85336	Cell Signaling Technology, USA
In vivo mAb anti-mouse PD-1	BE0146	Bio X Cell, USA
In vivo mAb anti-mouse CD4	S0B0690	Starter, China
In vivo mAb anti-mouse CD8	S0B0660	Starter, China
Anti-human/mouse STAT1	TP53449	Abmart, China
Anti-human/mouse p-STAT1(S727)	T55702	Abmart, China
Anti-human/mouse FAS	Abs131441	Absin, USA
Anti-human/mouse TRIM24	GXP513621	Genxspan, USA
Anti-human/mouse TRIM24	TA0320	Abmart, China
Anti-human/mouse caspase 3	T40044S	Abmart, China
Anti-human/mouse cleaved caspase 3 (D175)	TA7022	Abmart, China

(*Continued*)

(*Continued*)

Markers	No.	Company
Anti-human/mouse CXCL10	10937-1-AP	Proteintech, China

murine IL-2 (50 ng/ml) for 48 hours. Then, the cells were fixed with 2.5% glutaraldehyde overnight at 4°C, rinsed with PBS, and dehydrated in ethanol and acetone. Next, samples were embedded in Spurr resin and polymerized for 48 hours at 65°C. Last, 70-nm sections were stained with 2% uranyl acetate and lead citrate. After rinsing, sections were visualized using a FEI Tecnai G2 Spirit transmission electron microscope.

Seahorse bioanalyzer

OCR and proton efflux rate (PER) were measured using a Seahorse XFe96 extracellular flux analyzer (Agilent Technologies). After plate coating by poly-L-lysine, NC and *Hmgb2*-deficient $CD8^+$ T cells were seeded at a density of 100,000 cells per well into XFe96 microplates. Next, microplates were placed at 37°C in a CO_2-free incubator for 30 min. Then, measuring solution was gently added, and cells were cultured at 37°C for another 15 min. Last, OCR and PER were measured by a sensor probe on a Seahorse XFe96 analyzer according to the manufacturer's instructions. Seahorse XF cell glycolytic rate test kits and XF cell mito stress test kits were used for the assays. Wave software was used to analyze the data.

Multiplex immunofluorescence

Tumor samples were collected, embedded in paraffin, and sectioned. The slides were deparaffinized in xylene and hydrated in graded ethanol solutions. Then, the slides were incubated with AR buffer, and the heat-induced antigen retrieval was performed. A total of 3% H_2O_2 and 3% bovine serum albumin were used to block endogenous peroxidases and antigens. Next, several rounds of the multiplex immunofluorescence staining procedure were conducted including incubation with the primary antibody, followed by horseradish peroxidase–conjugated secondary antibody and fluorescent substrate deposition. Last, the nuclei were counterstained with 4′,6-diamidino-2-phenylindole (DAPI) (D1306, Thermo Fisher Scientific). Images of the slides were obtained by a scanner (Pannoramic MIDI, 3DHISTECH).

Mice

C57BL/6J and BALB/C nude mice were purchased from Shanghai Jihui Experimental Animal Breeding Co. Ltd. *Cd4*-Cre and *Hmgb2*$^{flox/flox}$ mice were purchased from Cyagen Biosciences (Suzhou) Inc. and were backcrossed to C57BL/6J for generations. *Hmgb2*$^{flox/flox}$;*Cd4*-Cre OT-I mice were derived by crossing *Hmgb2*$^{flox/flox}$;*Cd4*-Cre mice with OT-I mice by Cyagen Biosciences (Suzhou) Inc. All mice were maintained in specific pathogen–free facilities of Fudan University Laboratory Animal Center. All animal protocols were approved by Shanghai Medical Experimental Animal Care Committee and followed the guidelines of the National Academy of Sciences and the National Institutes of Health.

In vivo models and therapies

For the subcutaneous tumor model, Hepa1-6 and MC38 cells (2×10^6) were resuspended in 100 μl of PBS and injected subcutaneously into the right posterior axilla of C57BL/6J wild-type (WT) mice, BALB/C nude mice, *Cd4*-Cre C57BL/6J mice, and *Hmgb2*$^{flox/flox}$;*Cd4*-Cre C57BL/6J mice. Tumor growth was recorded by standard calipers every 2 to 3 days, and tumor volumes were calculated by the following formula: length × width × width × 0.5. For orthotopic HCC model, Hepa1-6 shCtrl and shHmgb2 cells (1×10^6) were injected into liver subcapsular of 6-week-old C57BL/6J mice using microsyringes. For spontaneous HCC model, 2 ml of PBS containing pCMV-SB13, pT3–EF1a–N90–β-catenin, pT3-EF1a-NRas-GV12, pX330-sgTP53, and pT3-EF1A-MYC-IRES-Luc plasmid (10 μg per mice) into each mouse by hydrodynamic tail-vein injection. Mice were humanely euthanized at ~3 weeks, and tumor tissues were harvested for weight measurement and further flow cytometry and immunohistochemistry analyses. For the treatment regime, PD-1 antibody (5 mg/kg) was intraperitoneally injected every 3 days. Tannic acid (10 mg/kg) was given once a day by oral administration. Mice received one dose of anti-CD8 or anti-CD4 antibody (10 mg/kg) or of isotype control antibody (10 mg/kg), before tumor inoculation, and bi-weekly thereafter. For CXCR3 inhibitor AMG487 treatments, mice received intraperitoneal injection with AMG487 (5 mg/kg of body weight) or vehicle every 48 hours for 3 weeks, starting 24 hours before the subcutaneous Hepa1-6 cell injection. All the treatments were performed 7 days after tumor model construction.

ChIP assay

ChIP assay was conducted according to the instructions of ChIP kit (Bes5001, BersinBio, China). Briefly, DNA and associated proteins on chromatin in cultured cells were cross-linked by 1% formaldehyde for 10 min at RT. Then, cell nucleus was lysed in lysis buffer (with protease inhibitors and dithiothreitol), and supernatant was collected after ultrasonic lysis for 15 min. Approximately 5 mg of HMGB2 (catalog no. Ab67282, Abcam), TRIM24 (catalog no. GXP513621, GenXspan), or control IgG (catalog no. B30010, Abmart) was incubated with 20 μl of protein A/G magnetic beads at 4°C overnight. Next, after washing and elution at 65°C for 30 min, the immunoprecipitated complex was de–cross-linked at 65°C for 6 hours and then incubated with EDTA and Proteinase K at 55°C for 2 hours. Last, tris-saturated phenol was added into the solution, and NaCl and absolute alcohol were used to precipitate DNA.

Dual luciferase reporter assay

Hepa1-6 cells were seeded into 24-well plates, and the confluence reached to ~70% after 24 hours of incubation. According to the manufacturer's instruction, cells were transiently cotransfected with pCDNA3.1(+)-Hmgb2 plasmid, pCDNA3.1(+)-Trim24 plasmid, siTrim24 plasmid, PGL3-Stat1 promoter WT or mutant reporter plasmid, or NC plasmid using GMTrans liposomal transfection reagent (Genomeditech). After 48 hours, firefly luciferase reporter gene assay kit (040502A, Genomeditech) was used to detect firefly

Table 2. Names and sequence of primers. F, forward; R, reverse.

Primers	Sequence (5′ → 3′)
ACTIN F	GGGAAATCGTGCGTGACATTAAG
ACTIN R	TGTGTTGGCGTACAGGTCTTTG
HMGB2 F	GGGTGAAATGTGGTCTGAGCAGTC
HMGB2 R	TCCTCCTCCTCCTCCTCATCTTCT
CXCL11 F	GTGTGCTACAGTTGTTCAAGGCTT
CXCL11 R	CCTTGCTTGCTTCGATTTGGGATT
CXCL10 F	AACTGTACGCTGTACCTGCATCAG
CXCL10 R	TCTTGATGGCCTTCGATTCTGGAT
CXCL9 F	TCTTGCTGGTTCTGATTGGAGTGC
CXCL9 R	TTTCTCGCAGGAAGGGCTTGG
ISG15 F	GCGACGAACCTCTGAGCATCCT
ISG15 R	CGAAGGTCAGCCAGAACAGGTC
ISG20 F	GGTGCTGTGCTGTACGACAAGTT
ISG20 R	TGTGTAGCCGCTCATGTCCTCTT
CCL5 F	CAGCAGTCGTCCACAGGTCAAG
CCL5 R	CAAGAGCAAGCAGAAACAGGCAAA
Actin F	GGCTACAGCTTCACCACCACAG
Actin R	GGAACCGCTCGTTGCCAATAGT
Hmgb2 F	GGGTGAGATGTGGTCTGAGCAATC
Hmgb2 R	CTTCCTCCTCTTCTTCCTCCTCCT
Keap1 F	TGCTCAACCGCTTGCTGTATGC
Keap1 R	TTCAACTGGTCCTGCCCATCGT
Ndufa1 F	CCACTGCGTACATCCACAAATTCA
Ndufa1 R	GCCCTTGGACACATAGTAGCGATT
Ndufb3 F	GCTGCTGGACATGGACATGAACA
Ndufb3 R	CACCGTTTCTAATGGCGTCCCTTC
Ndufs6 F	CAACAGCCTGTGAATGAGGTGGAG
Ndufs6 R	TAGTGATGGTGCTGCTTGAACTGC
Sdhb F	TCTACCGCTGCCACACCATCAT
Sdhb R	GCCAATGCTCGCTTCTCCTTGT
Sdhc F	CTAAGGAGGAGATGGAGCGGTTCT
Sdhc R	AACGGACAGTGCCATAGGAAGAGA
Sdhd F	CCTGCTCTGTGGTGGACTACTCT
Sdhd R	TGCCGACATCGTGGTAATTGAAGT
Uqcr10 F	CGCCTGTACTCCTTGCTGTTCC
Uqcr10 R	CCTCGTTGATGTGCTCGTAGATCG
Uqcrb F	ATCAAGCAAGTGGCTGGATGGTT
Uqcrb R	GGTCCTCAGGAAGCCTTCTTATGG
Uqcrq F	GGTCCTCAGGAAGCCTTCTTATGG
Uqcrq R	GACTGCTCAAACTCCTGGTTGCC
Nfe2l2 F	TCTTGGAGTAAGTCGAGAAGTGT
Nfe2l2 R	GTTGAAACTGAGCGAAAAAGGC
Tfam F	ATTCCGAAGTGTTTTTCCAGCA
Tfam R	TCTGAAAGTTTTGCATCTGGGT
Tfb1m F	CGGGAGATCATTAAGTTGTTCGG
Tfb1m R	GCCCAGGACCCACTTCATAAA
Nqo1 F	AGGATGGGAGGTACTCGAATC
Nqo1 R	AGGCGTCCTTCCTTATATGCTA
Hmox1 F	AAGCCGAGAATGCTGAGTTCA

(*Continued*)

(*Continued*)

Primers	Sequence (5′ → 3′)
Hmox1 R	GCCGTGTAGATATGGTACAAGGA
Gpx1 F	AGTCCACCGTGTATGCCTTCT
Gpx1 R	GAGACGCGACATTCTCAATGA
Txn1 F	CATGCCGACCTTCCAGTTTTA
Txn1 R	TTTCCTTGTTAGCACCGGAGA
Txnrd1 F	TGTTGCTGGCGGTAGGAAGAGA
Txnrd1 R	AGGATGTCACCGATGGCGTAGA
Pparg F	CGCCAAGGTGCTCCAGAAGATG
Pparg R	GGTGAAGGCTCATGTCTGTCTCTG
Stat1 F	GCCTCTCATTGTCACCGAAGAACT
Stat1 R	TTGGAGATCACCACGACAGGAAGA
Cxcl10 F	AACCCAAGTGCTGCCGTCATTT
Cxcl10 R	AGGCTCGCAGGGATGATTTCAAG
Cxcl11 F	GAACAGGAAGGTCACAGCCATAGC
Cxcl11 R	TCAACTTTGTCGCAGCCGTTACTC
Irf9 F	CAACATAGGCGGTGGTGGCAAT
Irf9 R	GGTGAGCAGCAGCGAGTAGTCT
Irf1 F	TGTGTCGTCAGCAGCAGTCTCT
Irf1 R	TTGCGGCTTCGGAGGTGGAA
Fas F	TACTGCGATTCTCCTGGCTGTGA
Fas R	GGCGATTTCTGGGACTTTGTTTCC
Ccl5 F	GACACCACTCCCTGCTGCTTTG
Ccl5 R	GCACACACTTGGCGGTTCCTT
Cxcl9 F	AGCCGAGGCACGATCCACTA
Cxcl9 R	AGGCAGGTTTGATCTCCGTTCTTC
Isg15 F	AGCCTCTGAGCATCCTGGTGAG
Isg15 R	CAGAACTGGTCTTCGTGGACTTGT
Isg20 F	GCCATGACCTGAAGCACGACTT
Isg20 R	CCGCCAGTTGTTCTGGATGTTCTT
Irf7 F	AAGGGTCACCACACTACACCATCT
Irf7 R	CTAGACAAGCACAAGCCGAGACTG
Irf5 F	ATGCCACCTCAGCCGTACAAGA
Irf5 R	TCCTCTTCCTCCTCCTCCTCTTCT
Tap1 F	GGACGCTGGAGACATGCTGTGT
Tap1 R	CGCTGTGCTGGCTATGGTGAGA
Ifit1 F	GGCTGGAGTGTGCTGAGATGGA
Ifit1 R	CTTGGCGATAGGCTACGACTGC
Trim24 F	TCCTGTCGTGGAGCAGAGTTCA
Trim24 R	TATTAAGCGTGGTGGCGGATGC
ChIP Stat1-RARE F	GGTACAAGAGGGAATGTGTG
ChIP Stat1-RARE R	TGTCTAGGCAGATACTCTGG
Stat1 TSS (−100)–200 F	GCTGATTGGCTGAGGCGGAA
Stat1 TSS (−100)–200 R	CTGGCTCTCACTCACTCACTGC

and *Renilla* luciferase activities and recorded using microplate reader (SpectraMax L, Molecular Devices).

SPR assay

Experiments were performed at 25°C on a BIAcore T200 using CM5 sensor chips, and data were analyzed using BIAcore T200 Evaluation software (GE Healthcare) following the manufacturer's instruction. Briefly, a cell on the CM5 sensor chip was activated with a mixture of 200 μM 1-ethyl-3-(3-dimethylaminopropyl)carbodiimide and 50 μM *N*-hydroxysuccinimide at 10 μl/min for 420 s. A total of 50 μl of protein by mixing with 180 μl of 10 mM sodium acetate solution (pH 5.0) was then immobilized on the surface of the cell for two repetitive runs. The cell was then blocked with 1 M ethanolamine. A neighboring aisle that served as a reference was similarly activated and blocked. Molecule stock solution was diluted to a series of concentrations in PBS and was flowed at 10 ml/min for 150 s in each run. At the end of each flow, cells were regenerated for 5 min with 10 mM glycine-HCl (pH 2.0) solution at 10 μl/min. Data from the sample cell were collected using BIAcore T200 Control software (v. 2.0, GE Healthcare) and were subtracted by those from the reference cell. Association and dissociation constants were obtained by global fitting of the data to a 1:1 Langmuir binding model using BIAcore T200 Evaluation software (v.2.0, GE Healthcare). Data were exported to Origin 7 software (v.7.0552, OriginLab) for generating the final figures.

Molecular docking assay

The three-dimensional structure of proteins is derived from molecular dynamics simulations. The HMGB2 protein was protonated and optimized by molecular operating environment (MOE) plugin "Quickprep." Protein and molecule structures were protonated under AMBER10: EHT force field. MOE plugin "Dock" was used to study the interaction between protein and molecules. In the docking process, the proteins were coarse grained, and the fast Fourier transform was used to search the interaction mode. The coarse-grained model and the side chain of the contact residues were optimized, retaining up to 300 conformations using London δ score function; then docking poses were further optimized using energy minimization; and the binding energies were calculated by GB/VI scoring function. Last, the top 10 docking poses were retained, along with the docking pose.

Cell toxicity assay

Cells (1000 cells per well) were seeded in 384-well plates (25 μl per well). In succession, 25 μl of compound solutions in different concentrations were added and incubated for 72 hours. After incubation, a 10% CCK-8 (Apexbio, K1018, Houston, Beyotime) solution in medium was administered and reincubated for 2 hours. The absorbance at 450 nm was measured. Cell viability inhibition rate was calculated as follows: % inhibition = $[1 - (OD_{sample} - OD_{blank}) / (OD_{control} - OD_{blank})] \times 100\%$.

Details of antibodies

Markers and article numbers of antibodies were shown in Table 1.

Primers

Details of primers were shown in Table 2.

Statistical analysis

Statistical analysis was performed using R software 4.3.0 (R Foundation, Vienna, Austria) and SPSS 22.0 (IBM, Armonk, NY, USA). Statistical significance was determined by unpaired or paired Student's *t* test, and one- and two-way analysis of variance (ANOVA) tests. Kaplan-Meier survival analysis with log-rank test was used for comparison of survival curves. Correlations between variables were analyzed by Pearson' correlation analysis. Differences were considered significant when $P < 0.05$. All experiments have been reproduced in at least three independent experiments, unless otherwise specified in the figure legends.

Supplementary Materials

This PDF file includes:
Figs. S1 to S12
Table S1

REFERENCES AND NOTES

1. H. Sung, J. Ferlay, R. L. Siegel, M. Laversanne, I. Soerjomataram, A. Jemal, F. Bray, Global cancer statistics 2020: GLOBOCAN estimates of incidence and mortality worldwide for 36 cancers in 185 countries. *CA Cancer J. Clin.* **71**, 209–249 (2021).
2. J. M. Llovet, R. K. Kelley, A. Villanueva, A. G. Singal, E. Pikarsky, S. Roayaie, R. Lencioni, K. Koike, J. Zucman-Rossi, R. S. Finn, Hepatocellular carcinoma. *Nat. Rev. Dis. Primers* **7**, 6 (2021).
3. J. M. Llovet, R. Pinyol, R. K. Kelley, A. El-Khoueiry, H. L. Reeves, X. W. Wang, G. J. Gores, A. Villanueva, Molecular pathogenesis and systemic therapies for hepatocellular carcinoma. *Nat. Cancer* **3**, 386–401 (2022).
4. S. Bagchi, R. Yuan, E. G. Engleman, Immune checkpoint inhibitors for the treatment of cancer: Clinical impact and mechanisms of response and resistance. *Annu. Rev. Pathol.* **16**, 223–249 (2021).
5. J. S. O'Donnell, M. W. L. Teng, M. J. Smyth, Cancer immunoediting and resistance to T cell-based immunotherapy. *Nat. Rev. Clin. Oncol.* **16**, 151–167 (2019).
6. D. S. Chen, I. Mellman, Elements of cancer immunity and the cancer-immune set point. *Nature* **541**, 321–330 (2017).
7. M. Ayers, J. Lunceford, M. Nebozhyn, E. Murphy, A. Loboda, D. R. Kaufman, A. Albright, J. D. Cheng, S. P. Kang, V. Shankaran, S. A. Piha-Paul, J. Yearley, T. Y. Seiwert, A. Ribas, T. K. McClanahan, IFN-γ–related mRNA profile predicts clinical response to PD-1 blockade. *J. Clin. Invest.* **127**, 2930–2940 (2017).
8. M. F. Maurer, K. E. Lewis, J. L. Kuijper, D. Ardourel, C. J. Gudgeon, S. Chandrasekaran, S. L. Mudri, K. N. Kleist, C. Navas, M. F. Wolfson, M. W. Rixon, R. Swanson, S. R. Dillon, S. D. Levin, Y. R. Kimbung, M. Akutsu, D. T. Logan, B. Walse, K. M. Swiderek, S. L. Peng, The engineered CD80 variant fusion therapeutic davoceticept combines checkpoint antagonism with conditional CD28 costimulation for anti-tumor immunity. *Nat. Commun.* **13**, 1790 (2022).
9. M. Philip, A. Schietinger, $CD8^+$ T cell differentiation and dysfunction in cancer. *Nat. Rev. Immunol.* **22**, 209–223 (2022).
10. D. S. Thommen, T. N. Schumacher, T cell dysfunction in cancer. *Cancer Cell* **33**, 547–562 (2018).
11. M. E. Bianchi, A. Agresti, HMG proteins: Dynamic players in gene regulation and differentiation. *Curr. Opin. Genet. Dev.* **15**, 496–506 (2005).
12. T. Y. Starkova, A. M. Polyanichko, T. O. Artamonova, A. S. Tsimokha, A. N. Tomilin, E. V. Chikhirzhina, Structural characteristics of high-mobility group proteins HMGB1 and HMGB2 and their interaction with DNA. *Int. J. Mol. Sci.* **24**, 3577 (2023).
13. J. H. Kwon, J. Kim, J. Y. Park, S. M. Hong, C. W. Park, S. J. Hong, S. Y. Park, Y. J. Choi, I. G. Do, J. W. Joh, D. S. Kim, K. Y. Choi, Overexpression of high-mobility group box 2 is associated with tumor aggressiveness and prognosis of hepatocellular carcinoma. *Clin. Cancer Res.* **16**, 5511–5521 (2010).
14. E. N. Neubert, J. M. DeRogatis, S. A. Lewis, K. M. Viramontes, P. Ortega, M. L. Henriquez, R. Buisson, I. Messaoudi, R. Tinoco, HMGB2 regulates the differentiation and stemness of exhausted $CD8^+$ T cells during chronic viral infection and cancer. *Nat. Commun.* **14**, 5631 (2023).
15. F. Tang, J. Li, L. Qi, D. Liu, Y. Bo, S. Qin, Y. Miao, K. Yu, W. Hou, J. Li, J. Peng, Z. Tian, L. Zhu, H. Peng, D. Wang, Z. Zhang, A pan-cancer single-cell panorama of human natural killer cells. *Cell* **186**, 4235–4251.e20 (2023).
16. B. Ruf, M. Bruhns, S. Babaei, N. Kedei, L. Ma, M. Revsine, M. R. Benmebarek, C. Ma, B. Heinrich, V. Subramanyam, J. Qi, S. Wabitsch, B. L. Green, K. C. Bauer, Y. Myojin, L. T. Greten, J. D. McCallen, P. Huang, R. Trehan, X. Wang, A. Nur, D. Q. Murphy Soika, M. Pouzolles, C. N. Evans, R. Chari, D. E. Kleiner, W. Telford, K. Dadkhah, A. Ruchinskas, M. K. Stovroff, J. Kang, K. Oza, M. Ruchirawat, A. Kroemer, X. W. Wang, M. Claassen, F. Korangy, T. F. Greten, Tumor-associated macrophages trigger MAIT cell dysfunction at the HCC invasive margin. *Cell* **186**, 3686–3705.e32 (2023).

17. Y. Lu, A. Yang, C. Quan, Y. Pan, H. Zhang, Y. Li, C. Gao, H. Lu, X. Wang, P. Cao, H. Chen, S. Lu, G. Zhou, A single-cell atlas of the multicellular ecosystem of primary and metastatic hepatocellular carcinoma. *Nat. Commun.* **13**, 4594 (2022).
18. Q. Zhang, Y. He, N. Luo, S. J. Patel, Y. Han, R. Gao, M. Modak, S. Carotta, C. Haslinger, D. Kind, G. W. Peet, G. Zhong, S. Lu, W. Zhu, Y. Mao, M. Xiao, M. Bergmann, X. Hu, S. P. Kerkar, A. B. Vogt, S. Pflanz, K. Liu, J. Peng, X. Ren, Z. Zhang, Landscape and dynamics of single immune cells in hepatocellular carcinoma. *Cell* **179**, 829–845.e20 (2019).
19. X. Wang, Q. He, H. Shen, A. Xia, W. Tian, W. Yu, B. Sun, TOX promotes the exhaustion of antitumor $CD8^+$ T cells by preventing PD1 degradation in hepatocellular carcinoma. *J. Hepatol.* **71**, 731–741 (2019).
20. G. Q. Zhu, Z. Tang, R. Huang, W. F. Qu, Y. Fang, R. Yang, C. Y. Tao, J. Gao, X. L. Wu, H. X. Sun, Y. F. Zhou, S. S. Song, Z. B. Ding, Z. Dai, J. Zhou, D. Ye, D. J. Wu, W. R. Liu, J. Fan, Y. H. Shi, $CD36^+$ cancer-associated fibroblasts provide immunosuppressive microenvironment for hepatocellular carcinoma via secretion of macrophage migration inhibitory factor. *Cell Discov.* **9**, 25 (2023).
21. Y. Zhang, X. Yu, R. Bao, H. Huang, C. Gu, Q. Lv, Q. Han, X. Du, X. Y. Zhao, Y. Ye, R. Zhao, J. Sun, Q. Zou, Dietary fructose-mediated adipocyte metabolism drives antitumor $CD8^+$ T cell responses. *Cell Metab.* **35**, 2107–2118.e6 (2023).
22. E. Maldonado, D. A. Rojas, F. Urbina, A. Solari, The use of antioxidants as potential co-adjuvants to treat chronic chagas disease. *Antioxidants* **10**, 1022 (2021).
23. X. Qian, X. Li, Z. Shi, X. Bai, Y. Xia, Y. Zheng, D. Xu, F. Chen, Y. You, J. Fang, Z. Hu, Q. Zhou, Z. Lu, KDM3A senses oxygen availability to regulate PGC-1α-mediated mitochondrial biogenesis. *Mol. Cell* **76**, 885–895.e7 (2019).
24. E. Crisman, P. Duarte, E. Dauden, A. Cuadrado, M. I. Rodriguez-Franco, M. G. Lopez, R. Leon, KEAP1-NRF2 protein-protein interaction inhibitors: Design, pharmacological properties and therapeutic potential. *Med. Res. Rev.* **43**, 237–287 (2023).
25. A. V. Ulasov, A. A. Rosenkranz, G. P. Georgiev, A. S. Sobolev, Nrf2/Keap1/ARE signaling: Towards specific regulation. *Life Sci.* **291**, 120111 (2022).
26. G. Q. Zhu, Z. Tang, T. H. Chu, B. Wang, S. P. Chen, C. Y. Tao, J. L. Cai, R. Yang, W. F. Qu, Y. Wang, Q. F. Zhao, R. Huang, M. X. Tian, Y. Fang, J. Gao, X. L. Wu, J. Zhou, W. R. Liu, Z. Dai, Y. H. Shi, J. Fan, Targeting SRSF1 improves cancer immunotherapy by dually acting on $CD8^+$ T and tumor cells. *Signal Transduct. Target. Ther.* **10**, 25 (2025).
27. J. Li, C. Wu, H. Hu, G. Qin, X. Wu, F. Bai, J. Zhang, Y. Cai, Y. Huang, C. Wang, J. Yang, Y. Luan, Z. Jiang, J. Ling, Z. Wu, Y. Chen, Z. Xie, Y. Deng, Remodeling of the immune and stromal cell compartment by PD-1 blockade in mismatch repair-deficient colorectal cancer. *Cancer Cell* **41**, 1152–1169.e7 (2023).
28. L. B. Ivashkiv, IFNγ: Signalling, epigenetics and roles in immunity, metabolism, disease and cancer immunotherapy. *Nat. Rev. Immunol.* **18**, 545–558 (2018).
29. F. De Benedetti, G. Prencipe, C. Bracaglia, E. Marasco, A. A. Grom, Targeting interferon-γ in hyperinflammation: Opportunities and challenges. *Nat. Rev. Rheumatol.* **17**, 678–691 (2021).
30. K. M. Aird, O. Iwasaki, A. V. Kossenkov, H. Tanizawa, N. Fatkhutdinov, B. G. Bitler, L. Le, G. Alicea, T. L. Yang, F. B. Johnson, K. I. Noma, R. Zhang, HMGB2 orchestrates the chromatin landscape of senescence-associated secretory phenotype gene loci. *J. Cell Biol.* **215**, 325–334 (2016).
31. J. Tisserand, K. Khetchoumian, C. Thibault, D. Dembele, P. Chambon, R. Losson, Tripartite motif 24 (Trim24/Tif1α) tumor suppressor protein is a novel negative regulator of interferon (IFN)/signal transducers and activators of transcription (STAT) signaling pathway acting through retinoic acid receptor alpha (Rarα) inhibition. *J. Biol. Chem.* **286**, 33369–33379 (2011).
32. X. Li, M. Lu, M. Yuan, J. Ye, W. Zhang, L. Xu, X. Wu, B. Hui, Y. Yang, B. Wei, C. Guo, M. Wei, J. Dong, X. Wu, Y. Gu, CXCL10-armed oncolytic adenovirus promotes tumor-infiltrating T-cell chemotaxis to enhance anti-PD-1 therapy. *Oncoimmunology* **11**, 2118210 (2022).
33. J. Tan, W. Fan, T. Liu, B. Zhu, Y. Liu, S. Wang, J. Wu, J. Liu, F. Zou, J. Wei, L. Liu, X. Zhang, J. Zhuang, Y. Wang, H. Lin, X. Huang, S. Chen, M. Kuang, J. Li, $TREM2^+$ macrophages suppress $CD8^+$ T-cell infiltration after transarterial chemoembolisation in hepatocellular carcinoma. *J. Hepatol.* **79**, 126–140 (2023).
34. M. H. Hung, J. S. Lee, C. Ma, L. P. Diggs, S. Heinrich, C. W. Chang, L. Ma, M. Forgues, A. Budhu, J. Chaisaingmongkol, M. Ruchirawat, E. Ruppin, T. F. Greten, X. W. Wang, Tumor methionine metabolism drives T-cell exhaustion in hepatocellular carcinoma. *Nat. Commun.* **12**, 1455 (2021).
35. C. B. Nava Lauson, S. Tiberti, P. A. Corsetto, F. Conte, P. Tyagi, M. Machwirth, S. Ebert, A. Loffreda, L. Scheller, D. Sheta, Z. Mokhtari, T. Peters, A. T. Raman, F. Greco, A. M. Rizzo, A. Beilhack, G. Signore, N. Tumino, P. Vacca, L. A. McDonnell, A. Raimondi, P. D. Greenberg, J. B. Huppa, S. Cardaci, I. Caruana, S. Rodighiero, L. Nezi, T. Manzo, Linoleic acid potentiates $CD8^+$ T cell metabolic fitness and antitumor immunity. *Cell Metab.* **35**, 633–650.e9 (2023).
36. L. S. Levine, K. J. Hiam-Galvez, D. M. Marquez, I. Tenvooren, M. Z. Madden, D. C. Contreras, D. O. Dahunsi, J. M. Irish, O. O. Oluwole, J. C. Rathmell, M. H. Spitzer, Single-cell analysis by mass cytometry reveals metabolic states of early-activated $CD8^+$ T cells during the primary immune response. *Immunity* **54**, 829–844.e5 (2021).
37. L. Zhang, P. Romero, Metabolic control of $CD8^+$ T cell fate decisions and antitumor immunity. *Trends Mol. Med.* **24**, 30–48 (2018).
38. M. Peng, N. Yin, S. Chhangawala, K. Xu, C. S. Leslie, M. O. Li, Aerobic glycolysis promotes T helper 1 cell differentiation through an epigenetic mechanism. *Science* **354**, 481–484 (2016).
39. Y. Pan, T. Tian, C. O. Park, S. Y. Lofftus, S. Mei, X. Liu, C. Luo, J. T. O'Malley, A. Gehad, J. E. Teague, S. J. Divito, R. Fuhlbrigge, P. Puigserver, J. G. Krueger, G. S. Hotamisligil, R. A. Clark, T. S. Kupper, Survival of tissue-resident memory T cells requires exogenous lipid uptake and metabolism. *Nature* **543**, 252–256 (2017).
40. C. Li, Y. P. Phoon, K. Karlinsey, Y. F. Tian, S. Thapaliya, A. Thongkum, L. Qu, A. J. Matz, M. Cameron, C. Cameron, A. Menoret, P. Funchain, J. M. Song, C. M. Diaz-Montero, B. Tamilselvan, J. B. Golden, M. Cartwright, A. Rodriguez, C. Bonin, A. Vella, B. Zhou, B. R. Gastman, A high OXPHOS CD8 T cell subset is predictive of immunotherapy resistance in melanoma patients. *J. Exp. Med.* **219**, e20202084 (2022).
41. Z. Wu, H. Huang, Q. Han, Z. Hu, X. L. Teng, R. Ding, Y. Ye, X. Yu, R. Zhao, Z. Wang, Q. Zou, SENP7 senses oxidative stress to sustain metabolic fitness and antitumor functions of $CD8^+$ T cells. *J. Clin. Invest.* **132**, e155224 (2022).
42. X. Zhai, K. Liu, H. Fang, Q. Zhang, X. Gao, F. Liu, S. Zhou, X. Wang, Y. Niu, Y. Hong, S. H. Lin, W. H. Liu, C. Xiao, Q. Li, N. Xiao, Mitochondrial C1qbp promotes differentiation of effector $CD8^+$ T cells via metabolic-epigenetic reprogramming. *Sci. Adv.* **7**, eabk0490 (2021).
43. J. Zhang, D. Huang, P. E. Saw, E. Song, Turning cold tumors hot: From molecular mechanisms to clinical applications. *Trends Immunol.* **43**, 523–545 (2022).
44. P. A. Ott, Y. J. Bang, S. A. Piha-Paul, A. R. A. Razak, J. Bennouna, J. C. Soria, H. S. Rugo, R. B. Cohen, B. H. O'Neil, J. M. Mehnert, J. Lopez, T. Doi, E. M. J. van Brummelen, R. Cristescu, P. Yang, K. Emancipator, K. Stein, M. Ayers, A. K. Joe, J. K. Lunceford, T-cell-inflamed gene-expression profile, programmed death ligand 1 expression, and tumor mutational burden predict efficacy in patients treated with pembrolizumab across 20 cancers: KEYNOTE-028. *J. Clin. Oncol.* **37**, 318–327 (2019).
45. J. M. Llovet, R. Lencioni, mRECIST for HCC: Performance and novel refinements. *J. Hepatol.* **72**, 288–306 (2020).
46. M.-X. Tian, W.-R. Liu, H. Wang, Y.-F. Zhou, L. Jin, X. F. Jiang, C. Y. Tao, Z. Tang, P. Y. Zhou, Y. Fang, W.-F. Qu, Z.-B. Ding, Y.-F. Peng, Z. Dai, S.-J. Qiu, J. Zhou, W. Y. Lau, J. Fan, Y.-H. Shi, Tissue-infiltrating lymphocytes signature predicts survival in patients with early/intermediate stage hepatocellular carcinoma. *BMC Med.* **17**, 106 (2019).
47. I. Korsunsky, N. Millard, J. Fan, K. Slowikowski, F. Zhang, K. Wei, Y. Baglaenko, M. Brenner, P. R. Loh, S. Raychaudhuri, Fast, sensitive and accurate integration of single-cell data with Harmony. *Nat. Methods* **16**, 1289–1296 (2019).
48. H. Tian, H. Liu, Y. Zhu, D. Xing, B. Wang, The trends of single-cell analysis: A global study. *Biomed. Res. Int.* **2020**, 7425397 (2020).
49. H. S. Kaya-Okur, S. J. Wu, C. A. Codomo, E. S. Pledger, T. D. Bryson, J. G. Henikoff, K. Ahmad, S. Henikoff, CUT&Tag for efficient epigenomic profiling of small samples and single cells. *Nat. Commun.* **10**, 1930 (2019).

Acknowledgments: The pattern diagram and figure templates were designed and permitted by BioRender (https://app.biorender.com/). The publication licenses were provided in each figure legend. **Funding:** This work was supported by the grants from the National Natural Science Foundation of China (nos. 882073217, 82073218, 82003084, 82273387, and 82273386), Shanghai Municipal Science and Technology Major Project (grant no. 2018SHZDZX05), Shanghai Municipal Key Clinical Specialty, CAMS Innovation Fund for Medical Sciences (CIFMS) (2019-I2M-5-058), and National Key R&D Program of China (2020YFE0202200, 2018YFF0301102, and 2018YFF0301105). **Author contributions:** Y.-H.S., Z.T., W.-R.L, J.Z and J.F. carried out the conception and supervision of the study. W.-F.Q., G.-Q.Z., R.Y., T.-H.C., Z.-Q.G., R.H., M.-X.T., X.-F.J., C.-Y.T., Y.F., and J.G. designed and performed the experiments. W.-F.Q., G.-Q.Z., R.Y., T.-H.C., and Z.-Q.G. analyzed and interpreted the data. X.-L.W., J.-F.C., Q.-F.Z., Y.W., and Y.-C.B. collected clinical samples and data. W.-F.Q., G.-Q.Z., R.Y., T.-H.C., and Z.-Q.G. wrote the manuscript with the revision. **Competing interests:** The authors declare that they have no competing interests. **Data and materials availability:** All data needed to evaluate the conclusions in the paper are present in the paper and/or the Supplementary Materials. The scRNA-seq data have been deposited in the Gene Expression Omnibus (GEO) (www.ncbi.nlm.nih.gov/geo/query/acc.cgi) with accession number GSE202642.

Submitted 1 September 2024
Accepted 27 March 2025
Published 2 May 2025
10.1126/sciadv.ads8597
