# Supplementary material for: Targeting HMGB2 acts as dual immunomodulator by bolstering CD8+ T cell function and inhibiting tumor growth in hepatocellular carcinoma
Source: Sci Adv. 2025 May 2;11(18):eads8597. doi: 10.1126/sciadv.ads8597 (PMC12047442; doi:10.1126/sciadv.ads8597)
Supplement: Supplementary file 2 — Figs. S1 to S12 Table S1 [file sciadv.ads8597_sm.pdf]

Science Advances
AAAS

Supplementary Materials for

Targeting HMGB2 acts as dual immunomodulator by bolstering $CD8^+$ T cell function and inhibiting tumor growth in hepatocellular carcinoma

Wei-Feng Qu *et al.*

Corresponding author: Ying-Hong Shi, shi.yinghong@zs-hospital.sh.cn;
Zheng Tang, tang.zheng@zs-hospital.sh.cn; Wei-Ren Liu, liu.weiren@zs-hospital.sh.cn

Sci. Adv. **11**, eads8597 (2025)
DOI: 10.1126/sciadv.ads8597

This PDF file includes:

Figs. S1 to S12
Table S1

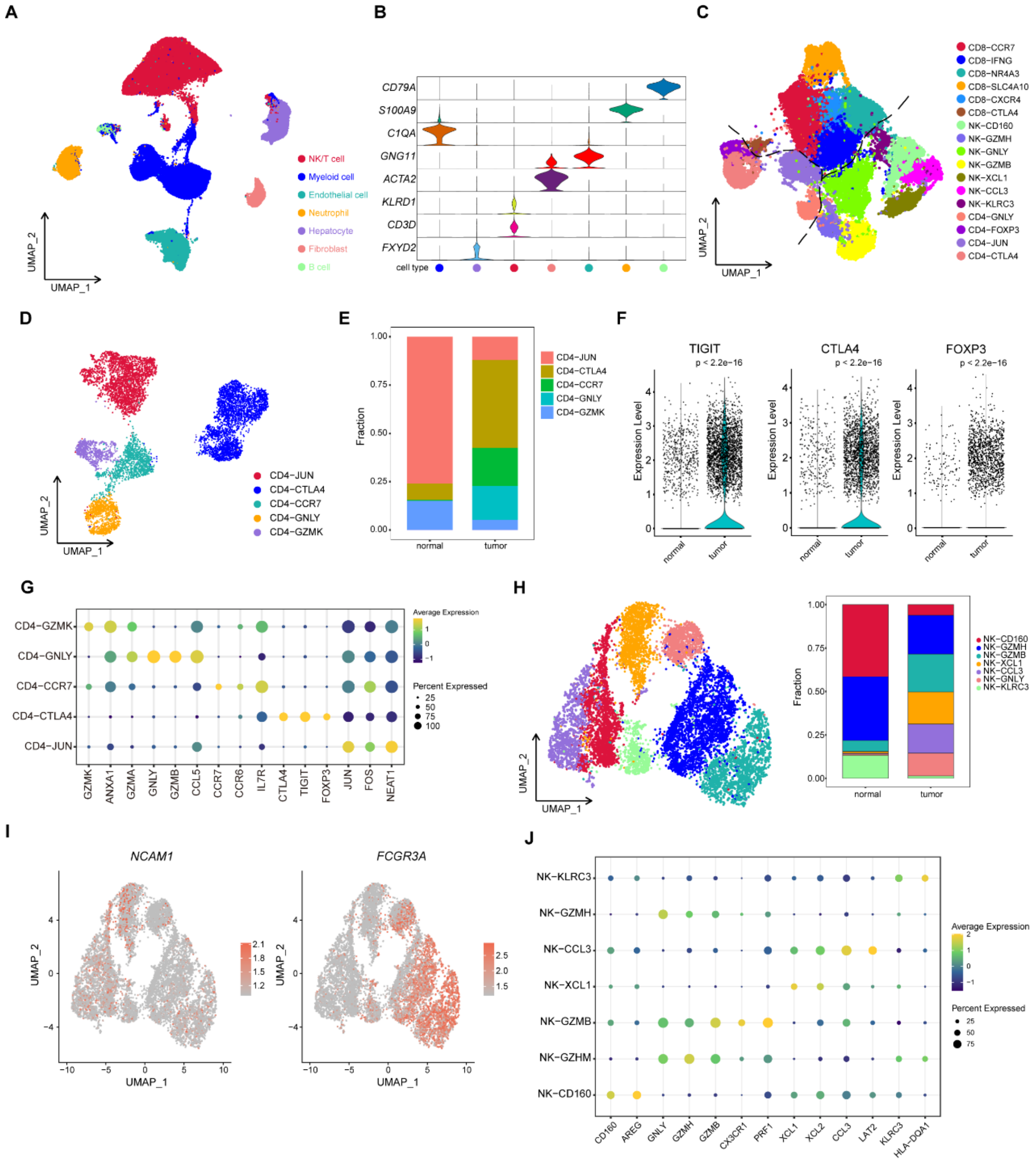

Figure S1. Single-cell transcriptome landscape of CD4 and NK cells. A. UMAP plot for the identification of different clusters in human HCC single-cell profiles. B. Violin plot shows the specific cell markers for different cell types in human HCC tissues. C. UMAP plot for the identification of subclusters in NK/T cell type. D. UMAP plot for the identification of subclusters in CD4^{+} T cells. E. Cell proportions of CD4^{+} T cells in tumor and normal liver tissues. F. Comparison of *TIGIT*, *CTLA4* and *FOXP3* expression of CD4^{+} T cells in normal liver and tumor tissues. G. Dot plot shows cell markers in different CD4^{+} T subclusters. H. UMAP plot and cell proportions of NK subclusters in Zhongshan cohort. I. UMAP plots show *NCAM1* and *FCGR3A* expression pattern in NK cells. J. Dot plot shows cell markers in different NK subclusters. Wilcoxon test for F.

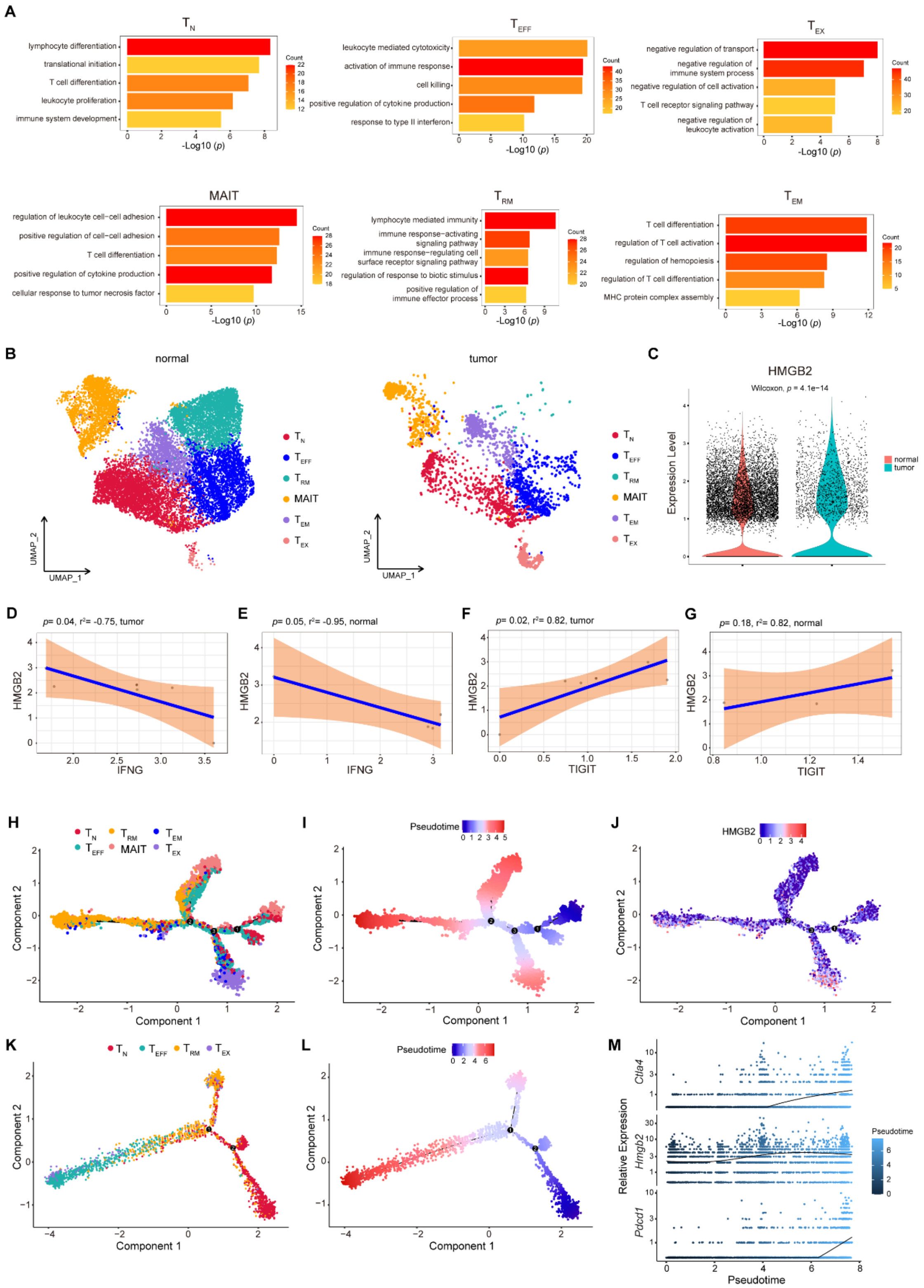

Figure S2. Heterogeneity of CD8$^+$ T cells at transcriptome level. A. Pathway enrichment of different subclusters in human CD8$^+$ T cells based on KEGG analysis. B. UMAP plots show different cell distribution of CD8$^+$ T cells in normal liver and tumor tissues. C. Comparison of HMGB2 expression in CD8$^+$ T cells in normal liver and tumor tissues. D. Correlation analysis of *HMGB2* and *IFNG* expression in CD8$^+$ T cells

of tumor tissues in Zhongshan cohort. E. Correlation analysis of *HMGB2* and *IFNG* expression in CD8^{+} T cells of normal liver tissues in Zhongshan cohort. F. Correlation analysis of *HMGB2* and *TIGIT* in intratumoral CD8^{+} T cells. G. Correlation analysis of *HMGB2* and *TIGIT* expression in CD8^{+} T cells of normal liver tissues. H. Trajectory plot shows the cell types of CD8^{+} T cells in human HCC tissues. I. Trajectory plot shows the pseudo-time of CD8^{+} T cells in human HCC tissues. J. Trajectory plot shows HMGB2 expression of CD8^{+} T cells in human HCC tissues. K. Trajectory plot shows the cell types of CD8^{+} T cells in mouse HCC tissues. L. Trajectory plot shows the pseudo-time of CD8^{+} T cells in mouse HCC tissues. M. Dotplot shows gene expression evolution of CD8^{+} T cells in mouse HCC tissues. Wilcoxon test for C. Pearson test for D, E, F, G. KEGG: Kyoto Encyclopedia of Genes and Genomes; T_{RM}, resident memory T cells; T_{EFF}, effector T cells; T_{N}, naïve T cells; T_{EM}, effective memory T cells; MAIT, mucosal-associated invariant T cell; T_{EX}, exhausted T cells.

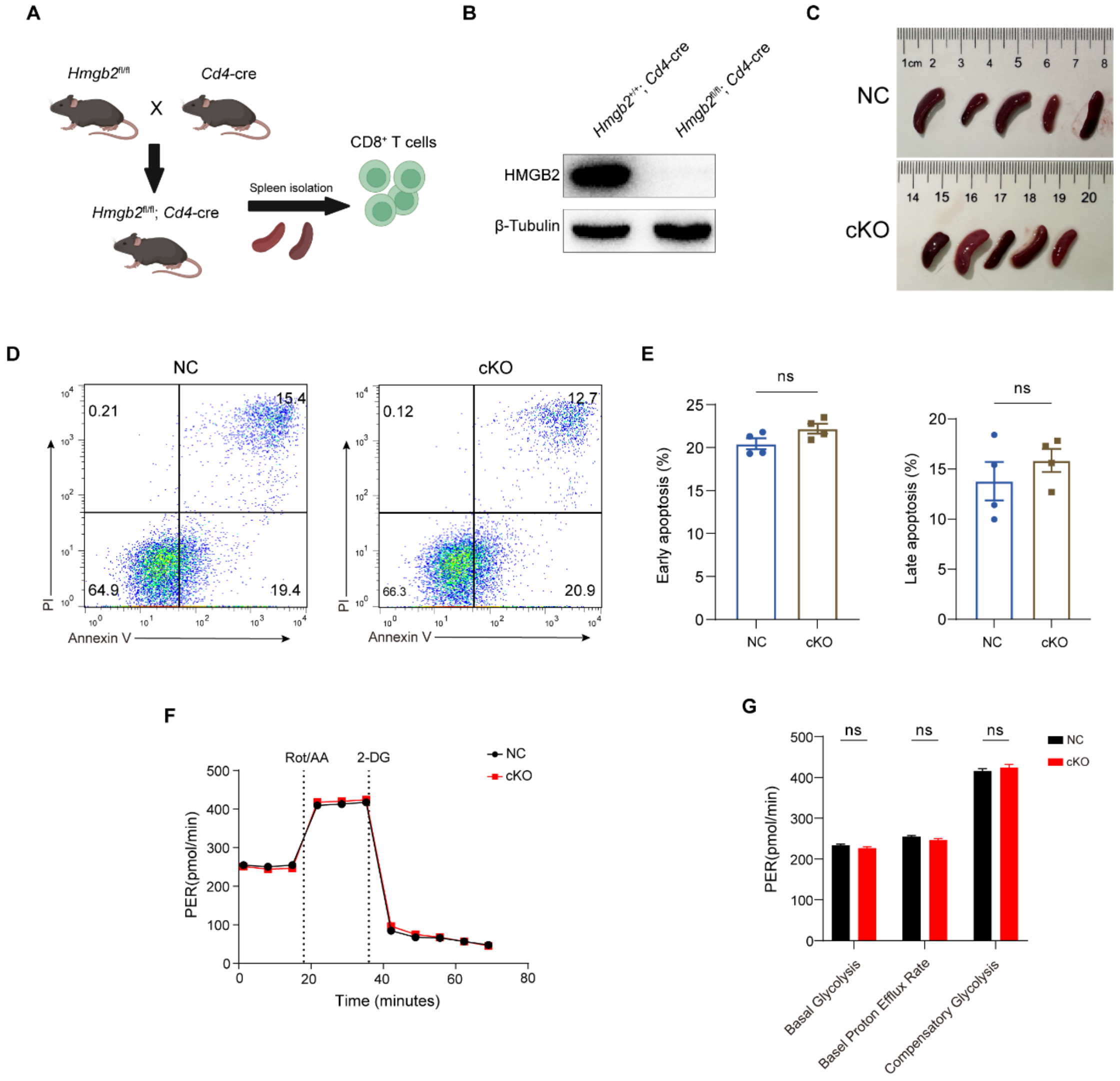

Figure S3. HMGB2 deficiency does not affect glycolysis. A. Schematic procedure for construction of *Hmgb2* conditional knockout mouse. B. Protein changes of HMGB2 in CD8^{+} T cells of *Hmgb2*$^{fl/fl}$; *Cd4*-cre mouse. C. Images of spleens in NC and cKO mice.

D. Annexin/V apoptosis analysis for NC and *Hmgb2*-cKO CD8$^+$ T cells. E. Quantification for apoptotic proportions of NC and *Hmgb2*-cKO CD8$^+$ T cells. F. Seahorse extracellular flux analysis of PER in activated NC and *Hmgb2*-cKO CD8$^+$ T cells. G. Quantification of seahorse extracellular flux analysis of PER (n=37). Data are presented as the mean ± SEM. ns, no significance. Student's t test for E. Two-way ANOVA test for G. NC, negative control; cKO, *Hmgb2* conditional knockout; PER, proton efflux rate. Fig.S3A is created in BioRender. Qu, W. (2025) https://BioRender.com/i17j890

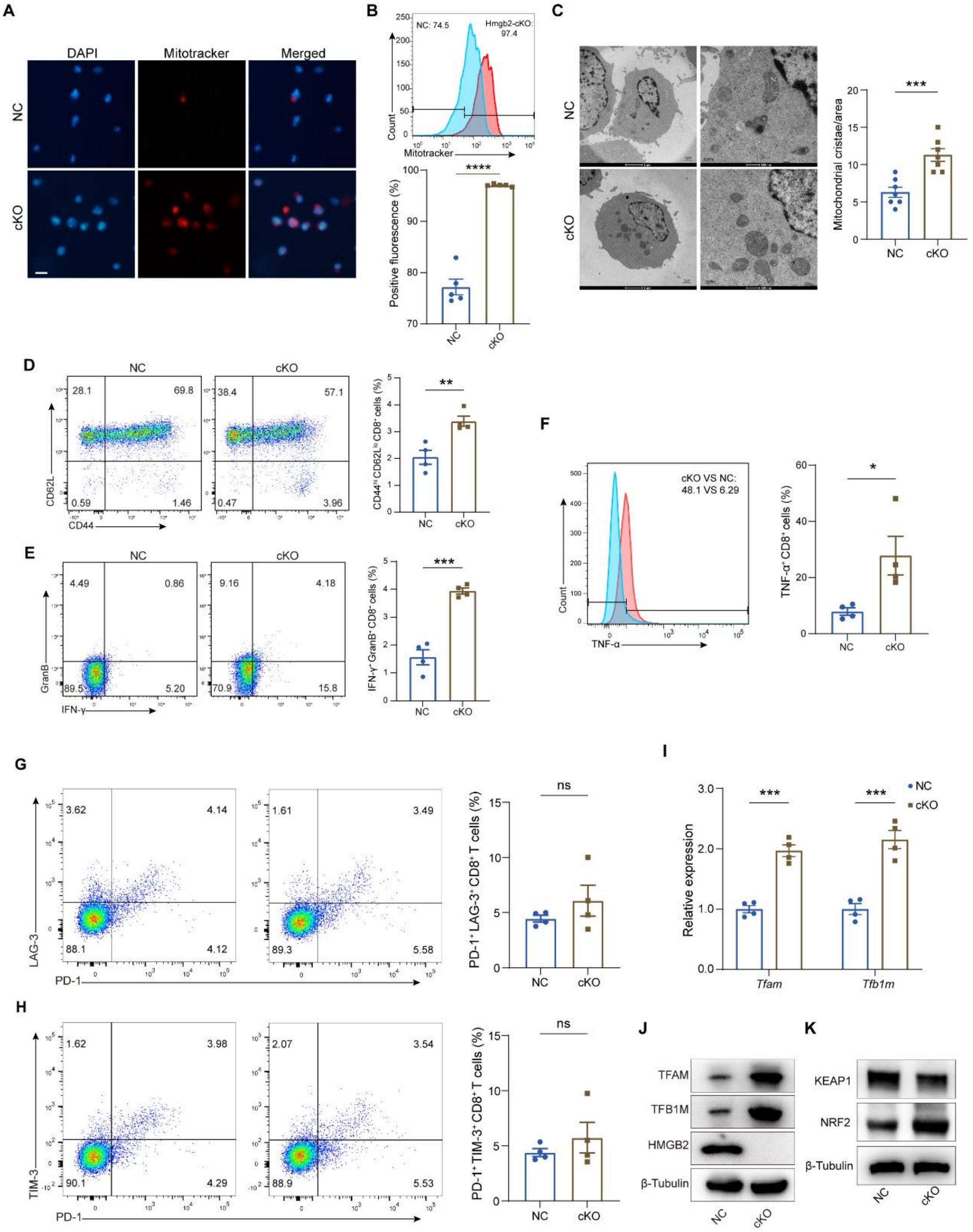

Figure S4. *Hmgb2* deficiency enhances mitochondrial oxidative phosphorylation

in splenic CD8$^+$ T cells. A. Immunofluorescence micrographs of splenic NC and *Hmgb2*-cKO CD8$^+$ T cells stained with MitoTracker (red), and 4′,6-diamidino-2-phenylindole (DAPI) (blue). Scale bars, 10 μm. B. Flow cytometry shows the positive fluorescence of stained MitoTracker in splenic NC and *Hmgb2*-cKO CD8$^+$ T cells (n=5). C. Transmission electron microscope images of mitochondria in isolated NC and *Hmgb2*-cKO CD8$^+$ T cells. The density of mitochondrial cristae is compared (n=7). D. Flow cytometry and quantification of splenic CD44^{hi} CD62L^{lo} effector CD8$^+$ T cells from NC and *Hmgb2*-cKO mice (6 to 8 weeks old, n= 4). E. Flow cytometry and quantification of splenic GranB$^+$ IFN-γ$^+$ CD8$^+$ T cells as in (D). F. Flow cytometry and quantification of splenic TNF-α$^+$ CD8$^+$ T cells as in (D). G. Flow cytometry and quantification of splenic PD1$^+$ LAG-3$^+$ CD8$^+$ T cells as in (D). H. Flow cytometry and quantification of splenic PD1$^+$ TIM-3$^+$ CD8$^+$ T cells as in (D). I. Comparison of *Tfam* and *Tfb1m* mRNA expression in isolated NC and *Hmgb2*-cKO CD8$^+$ T cells. J. Protein changes of TFAM and TFB1M in NC and *Hmgb2*-cKO CD8$^+$ T cells. K. Protein changes of KEAP1 and NRF2 in NC and *Hmgb2*-cKO CD8$^+$ T cells. Data are presented as the mean ± SEM. ns, no significance; *p< 0.05; **p< 0.01; ***p< 0.001; ****p< 0.0001. Student's t test. NC, negative control; cKO, *Hmgb2* conditional knockout.

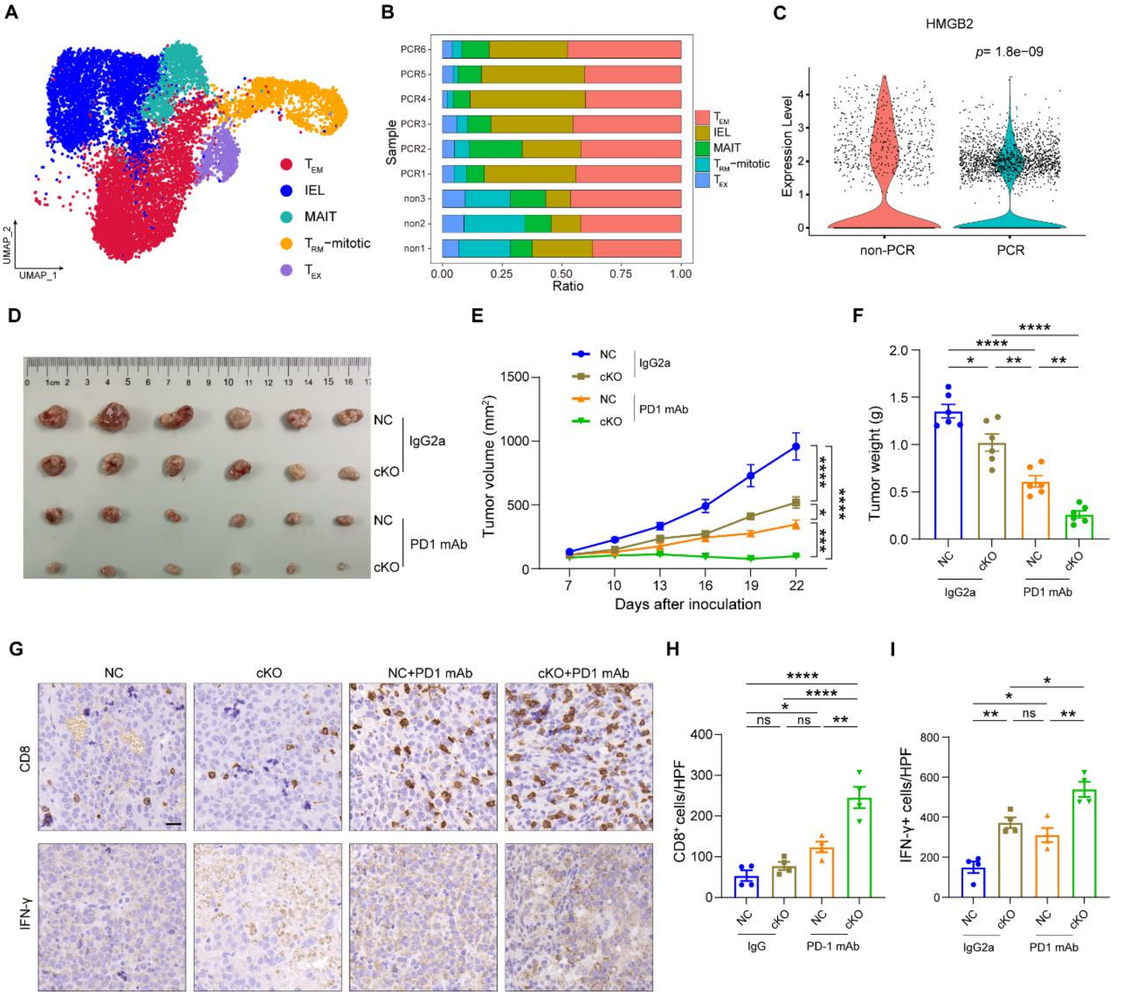

Figure S5. HMGB2 deficiency attenuates HCC tumor growth and improves the

efficacy of anti-PD-1 immunotherapy. A. UMAP plot for the different subclusters of $CD8^+$ T cells in GSE205506 dataset. B. Different cell proportions of $CD8^+$ T cells in PCR and non-PCR patients receiving neoadjuvant ICIs treatment in GSE205506 dataset. C. Comparison of HMGB2 expression of $CD8^+$ T cells in PCR and non-PCR patients in GSE205506 dataset. D. Tumor representative images of the subcutaneous HCC models in NC and *Hmgb2*-CKO mice (n= 6). E. Tumor growth curve of the subcutaneous HCC models (n= 6). F. Comparison for the tumor weights of the harvested subcutaneous HCC lesions (n= 6). G. Representative images of the immunohistochemistry staining of CD8 and IFN-γ in subcutaneous HCC tissues. Scale bar, 20 μm. H. Comparison of $CD8^+$ staining number in the subcutaneous HCC tissues. I. Comparison of IFN-γ^+ staining number in the subcutaneous HCC tissues. Data are presented as the mean ± SEM. ns, no significance; *$p< 0.05$; **$p< 0.01$; ***$p< 0.001$; ****$p< 0.0001$. Wilcoxon test for C. Two-way ANOVA test for E. One-way ANOVA tests for F, H and I. NC, negative control; cKO, *Hmgb2* conditional knockout; PCR, pathological complete response; mAb, mono-antibody.

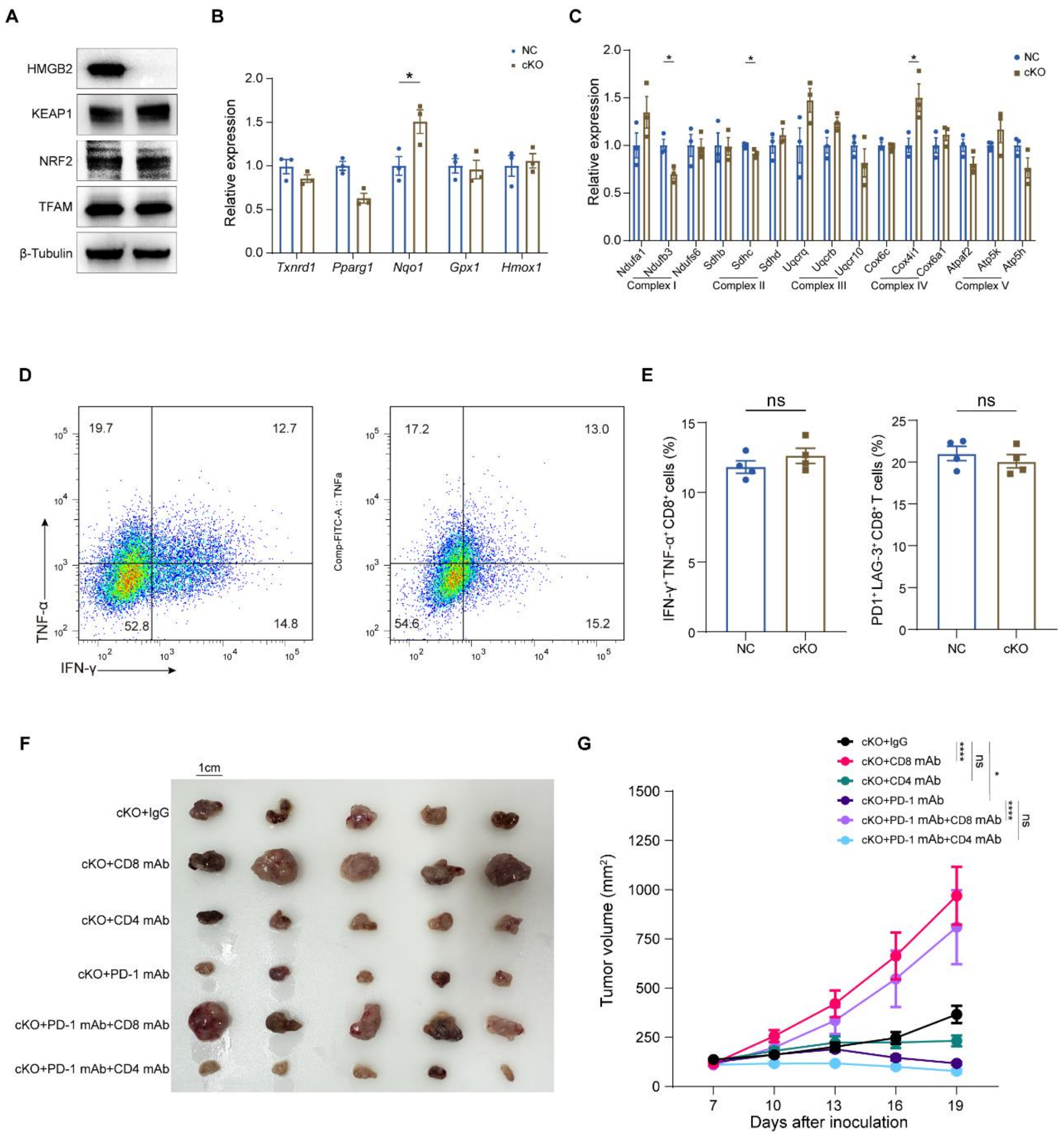

Figure S6. ***Hmgb2*** **deficiency in CD4⁺ T cells does not affect anti-tumor cytotoxicity of CD8⁺ T cells.** A. Western blotting experiment shows pathway changes in CD4⁺ T cells after *Hmgb2* knockout. B. mRNA levels of antioxidant response element genes in NC and *Hmgb2*-cKO CD4⁺ T cells (n= 3). C. mRNA levels of electron transport chain genes in NC and *Hmgb2*-cKO CD4⁺ T cells (n= 3). D. Flow cytometry analysis of OT-I CD8⁺ cells after coculture with Hepa 1-6 OVA cells and NC or *Hmgb2*-cKO CD4⁺ T cells. E. Quantification of IFN-γ⁺ TNF-α⁺ CD8⁺ T cells and PD1⁺ LAG-3⁺ CD8⁺ T cells as in (D) (n=4). F. Representative images of subcutaneous HCC tumors constructed by Hepa1-6 cell injection using *Hmgb2*-cKO mice. G. Tumor growth curves of subcutaneous tumors as in (F) (n= 5). Data are presented as the mean ± SEM. ns, no significance; *$p < 0.05$; ****$p < 0.0001$. Student's t test for B, C and E. Two-way ANOVA test for G. NC, negative control; cKO, *Hmgb2* conditional knockout; OVA, ovalbumin. mAb, mono-antibody.

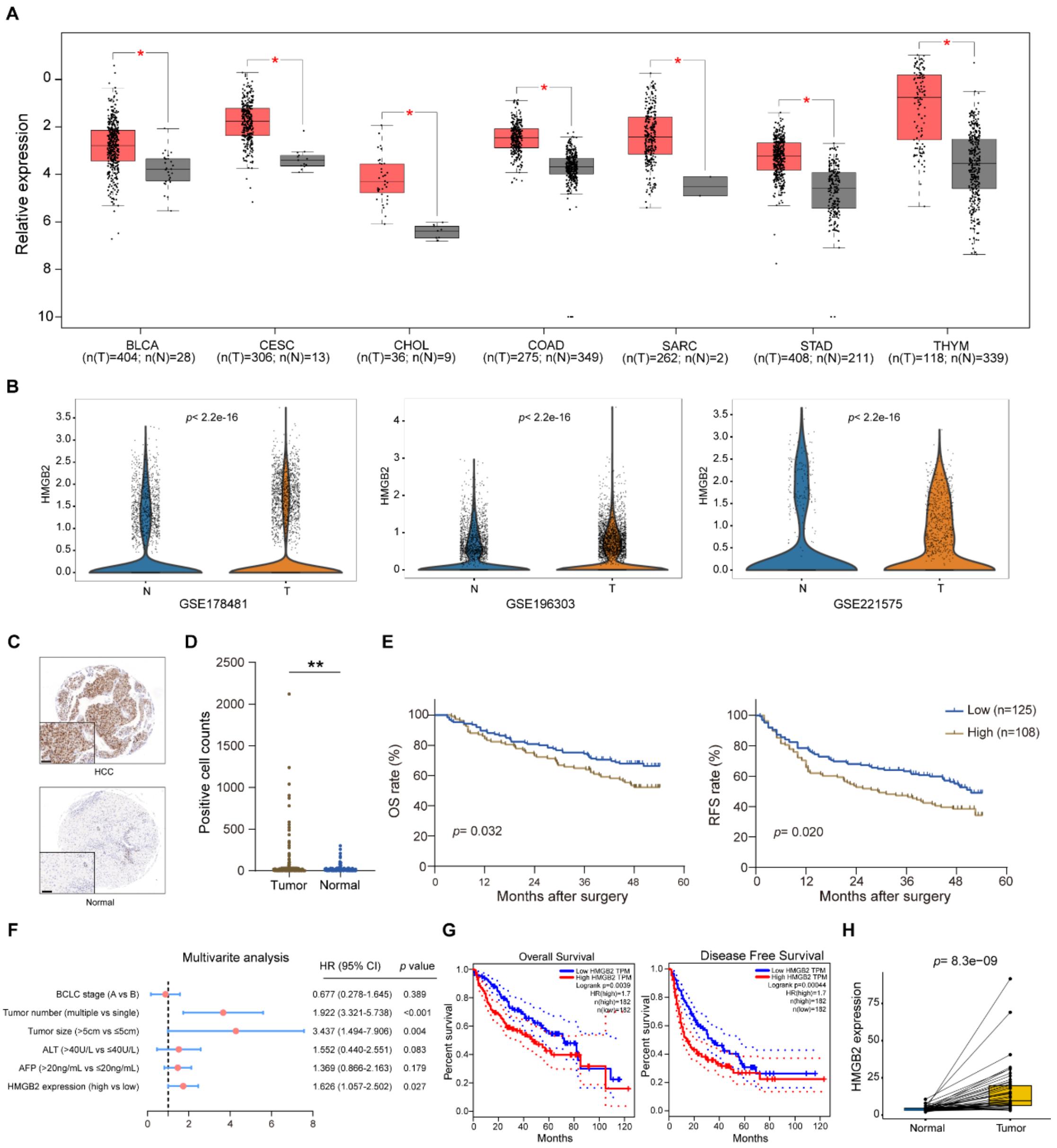

Figure S7. HMGB2 expression correlates with worse prognosis of HCC patients. A. mRNA levels of *HMGB2* in different solid tumors from TCGA database. B. Comparison of *HMGB2* expression in tumor cells and normal epithelial cells based on scRNA-seq datasets regarding clear cell renal cell carcinoma (GSE178481), lung carcinoid tumors (GSE196303) and colorectal cancer (GSE221575). C. Representative images for the immunohistochemistry staining of HMGB2 in tumor and normal liver tissue. Scale bar, 100 μm. D. Comparison of HMGB2 expression in tumor and normal liver tissue. E. The association of HMGB2 expression with OS and RFS of patients with HCC. F. Multivariate analysis for OS of patients with HCC. G. Kaplan–Meier survival curves reveal correlation between HMGB2 expression and OS and DFS based on TCGA-LIHC dataset. H. Comparison of HMGB2 mRNA levels of paired tumor and normal liver tissues based on TCGA-LIHC dataset. Data are presented as the mean ± SEM. ns, no significance; *$p< 0.05$; **$p< 0.01$. Wilcoxon test for A, B and H. Student's t test for D. Log-rank test for E and G. BLCA, bladder urothelial carcinoma; CESC,

cervical squamous cell carcinoma and endocervical adenocarcinoma; CHOL, cholangiocarcinoma; COAD, colorectal adenocarcinoma; SARC, sarcoma; STAD, stomach adenocarcinoma; THYM, thymoma; LIHC, liver hepatocellular carcinoma; OS, overall survival; RFS, recurrence free survival; DFS, disease free survival; ALT, alanine aminotransferase; AFP, α-fetoprotein.

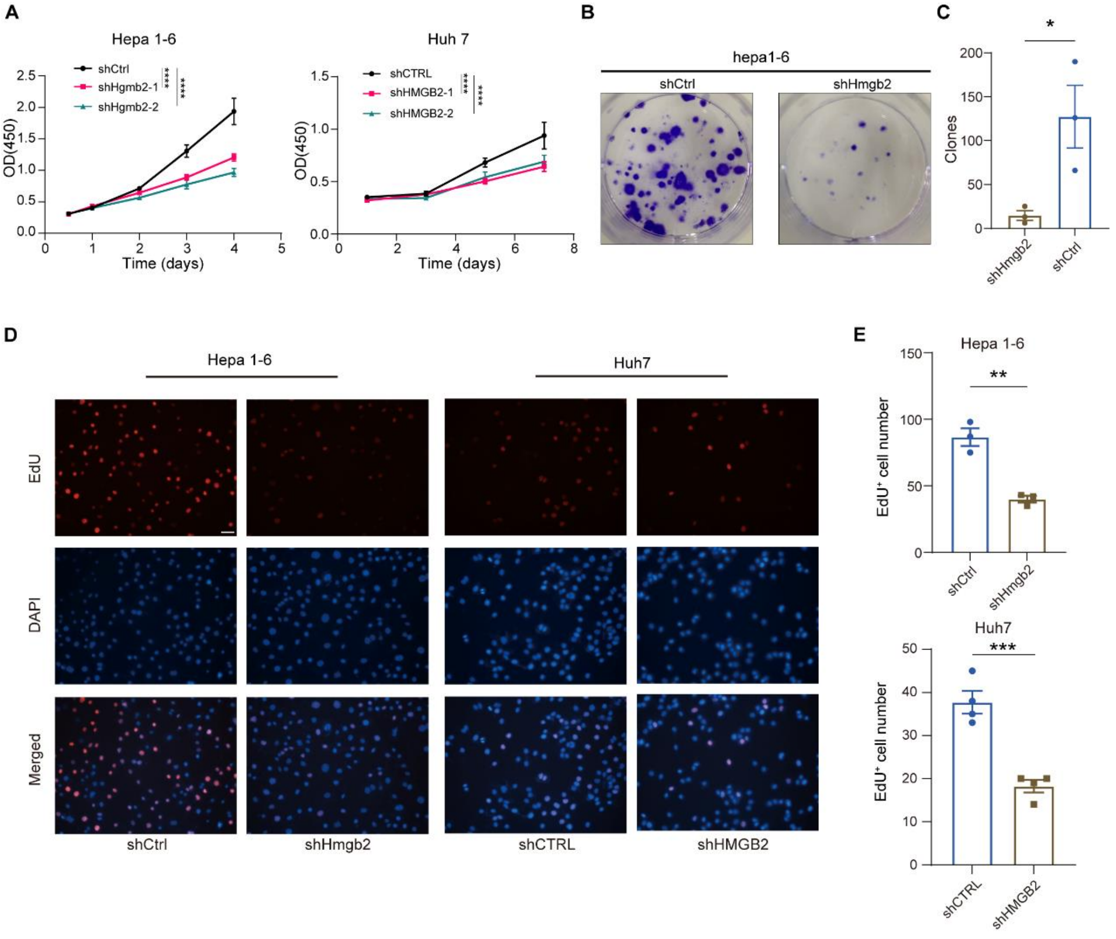

Figure S8. HMGB2 enhances proliferation of HCC cells. A. CCK8 proliferation curves for Hepa1-6 and Huh7 cell lines. B. Representative images for cell clone staining of Hepa1-6 cell lines. C. Comparison for clone numbers of Hepa1-6 cell lines. D. Representative images for EdU staining of Hepa1-6 and Huh7 cells. E. Comparison for EdU^+ cell numbers of Hepa1-6 and Huh7 cells. Data are presented as the mean ± SEM. ns, no significance; *$p < 0.05$; **$p < 0.01$; ***$p < 0.001$; ****$p < 0.0001$. Two-way ANOVA test for A. Student's t test for C and E.

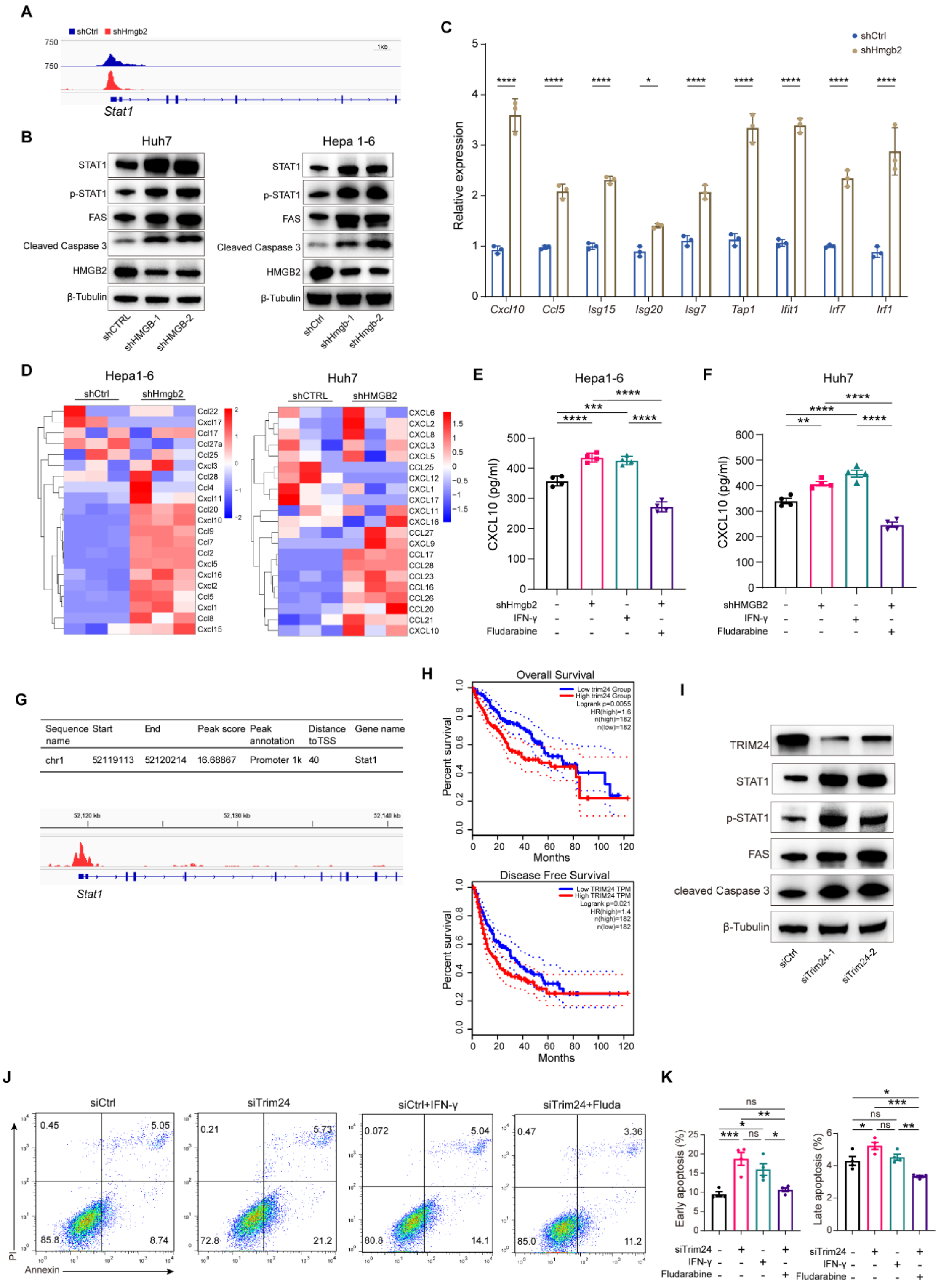

Figure S9. HMGB2 inhibits interferon-γ response via TRIM24/STAT1 axis. A. ATAC-seq tracks of *Stat1* in Hepa1-6 cells. B. Protein changes of STAT1 pathway in cells with differing HMGB2 expression levels. C. mRNA changes of ISGs in Hepa1-6 shCtrl and shHmgb2 cells (n= 3). D. Heatmaps of chemokine levels in cells with differing HMGB2 expression based on RNA-seq. E. ELISA assay of CXCL10 secretion in Hepa1-6 shCtrl and shHmgb2 cell lines. Cells were treated with IFN-γ (10 ng/mL) or Fludarabine (10 μM) for 24 hours (n= 4). F. ELISA assay of CXCL10 secretion in

Huh7 shCTRL and shHMGB2 cells. Cells were treated with IFN-γ (10 ng/mL) or Fludarabine (10 μM) for 24 hours (n= 4). G. CUT&Tag-seq reveals HMGB2 binding peak at promoter of *Stat1*. H. Kaplan–Meier survival curves reveal correlation between *TRIM24* expression and overall survival and disease-free survival based on TCGA-LIHC dataset. I. Protein changes of STAT1 pathway in Hepa1-6 cells with differing TRIM24 expression levels. J. Annexin/V apoptosis analysis for Hepa1-6 cells with differing TRIM24 expression levels. Cells were treated with IFN-γ (10 ng/mL) or Fludarabine (10 μM) for 24 hours. K. Quantification analysis for proportions of apoptotic Hepa1-6 cells with differing TRIM24 expression levels. Data are presented as the mean ± SEM. ns, no significance; *$p < 0.05$; **$p < 0.01$; ***$p < 0.001$; ****$p < 0.0001$. Student's t test for C. One-way ANOVA tests for E, F and K. ISGs, interferon stimulated genes.

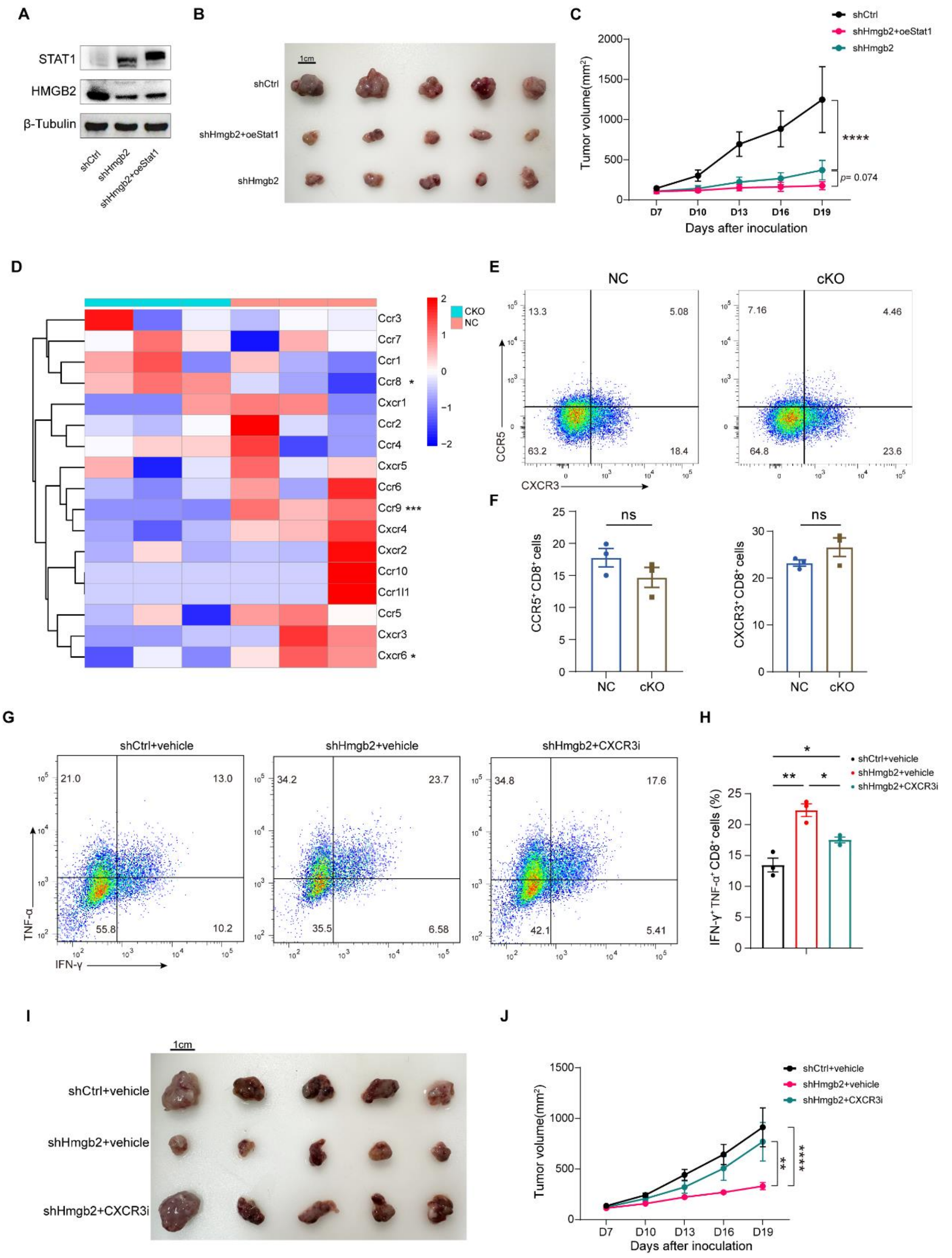

Figure S10. *Hmgb2* deficiency enhances anti-tumor immunity through CXCL10/CXCR3 signal. A. Western blotting experiment shows transfection effect of *Stat1* overexpression lentivirus. B. Representative images of subcutaneous HCC tumors constructed by shCtrl, shHmgb2 or shHmgb2+oeStat1 Hepa1-6 cell injection. Scale bar, 1cm. C. Tumor growth curves of subcutaneous tumors as in (F) (n= 5). D. Heatmap of chemokine receptor levels in $CD8^+$ T cells with differing HMGB2 expression based on RNA-seq. E. Flow cytometry analysis of chemokine receptors expressed on OT-I $CD8^+$ T cells after coculture with Hepa 1-6-OVA cells. F. Quantification of chemokine

receptors expressed on OT-I $CD8^+$ T cells as in (E). G. Flow cytometry analysis of OT-I $CD8^+$ T cells after coculture with shCtrl or shHmgb2 Hepa 1-6-OVA cells. CXCR3i (AMG487) was given at a concentration of 1 μmol/L. H. Quantification of IFN-γ^+ TNF-α^+ $CD8^+$ T cells as in (G) (n=3). I. Representative images of subcutaneous HCC tumors constructed by shCtrl or shHmgb2 Hepa1-6 cell injection. CXCR3i (AMG487) was intraperitoneally given at a concentration of 5 mg/kg every three days. Scale bar, 1cm (n=5). J. Tumor growth curves of subcutaneous tumors as in (I) (n= 5). Data are presented as the mean ± SEM. ns, no significance; *$p< 0.05$; **$p< 0.01$; ****$p< 0.0001$. Student's t test for D and F. Two-way ANOVA test for C and J. One-way ANOVA test for H.

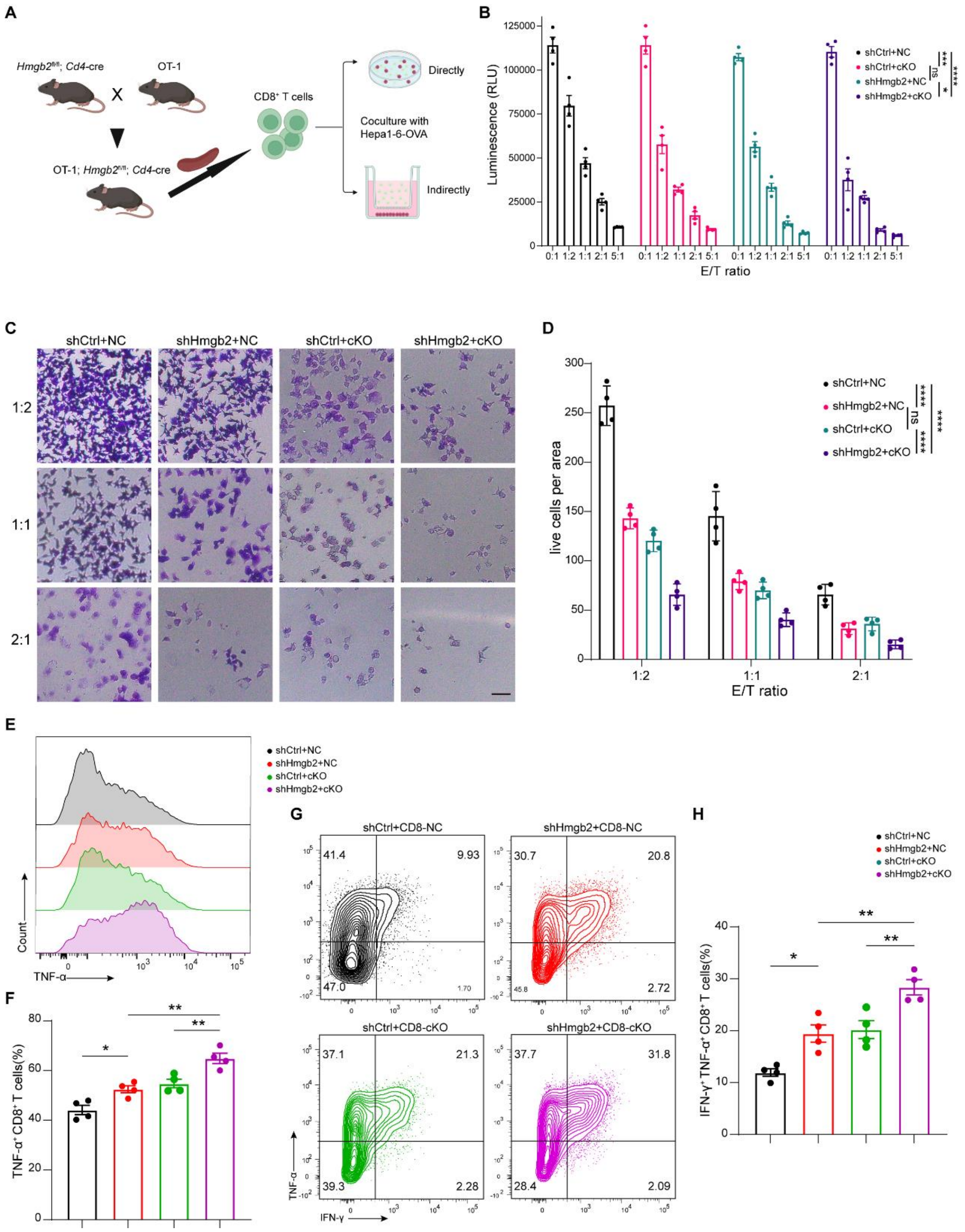

Figure S11. Dual HMGB2 inhibition boosts IFN-γ mediated T cell cytotoxicity. A. Schematic procedure for coculturing OT-I CD8^{+} T cells with Hepa1-6 cells expressing OVA. B. T cell killing assay with OT-I CD8^{+} T cells and Hepa1-6 cells expressing OVA (n= 4). Numbers of surviving tumor cells were quantified after 24 hours of direct coculture. C. Representative images for crystal violet staining of live Hepa1-6 cells after direct coculture with OT-I CD8^{+} T cells for 24 hours at different E/T ratio. Scale bar, 200 μm. D. Comparison for numbers of crystal violet stained cells as in (C) (n= 4). E. Flow cytometry of TNF-α expression of OT-I CD8^{+} T cells after indirect coculture with Hepa1-6 cells expressing OVA. F. Comparison for the proportions of TNF-α^{+}

CD8$^+$ T cells as in (E) (n= 4). G. Flow cytometry of TNF-α and IFN-γ in OT-I CD8$^+$ T cells as in (E). H. Comparison for the proportions of TNF-α$^+$ IFN-γ$^+$ CD8$^+$ T cells as in (E) (n= 4). Data are presented as the mean ± SEM. ns, no significance; *p< 0.05; **p< 0.01; ***p< 0.001; ****p< 0.0001. Two-way ANOVA test for B and D. One-way ANOVA test for F and H. E/T ratio, effector/target ratio. Fig.S11A is created in BioRender. Qu, W. (2025) https://BioRender.com/n96n391.

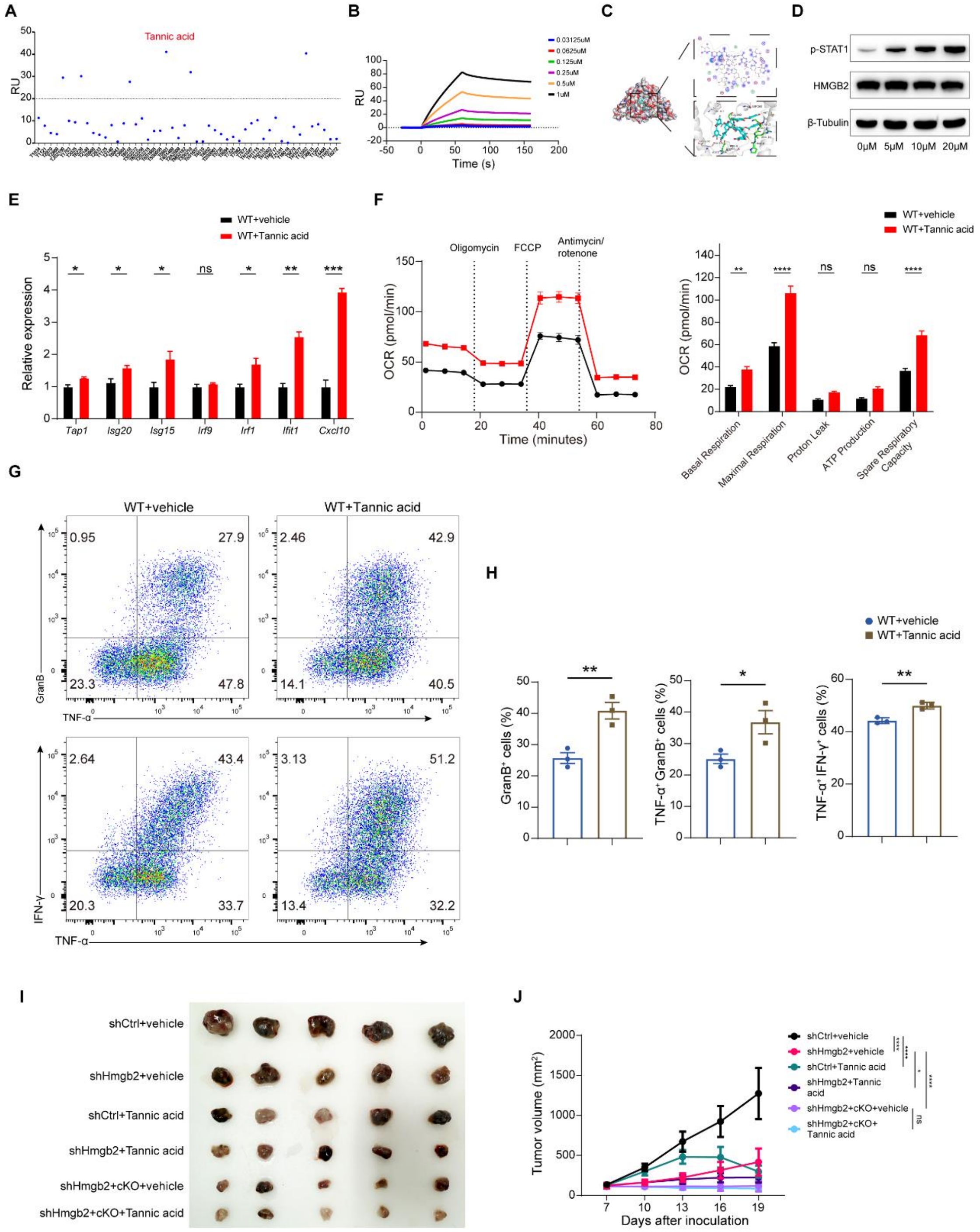

Figure S12. Tannic acid inhibits HMGB2 mediated pathway changes *in vitro*. A.

SPR binding levels for multiple compounds interacting with murine HMGB2 protein. Drug concentration, 100 μM. B. Binding affinity of tannic acid with murine HMGB2 protein under different drug concentration. C. 2D and 3D binding diagrams for amino acid structures of tannic acid with murine HMGB2 protein. D. Proteins changes of p-STAT1 and HMGB2 in Hepa1-6 cells after stimulation of tannic acid in different drug concentration. E. mRNA changes of ISGs in Hepa1-6 cells after stimulation of tannic acid (n= 3). Drug concentration, 20 μM for 24 hours. F. Seahorse extracellular flux analysis of OCR in WT and tannic acid-treated $CD8^+$ T cells (n= 8). Drug concentration, 8 μM for 24 hours. G. Flow cytometry of GranB, IFN-γ and TNF-α in WT and tannic acid-treated $CD8^+$ T cells. Drug concentration, 8 μM for 24 hours. H. Comparison of $GranB^+$ cells, TNF-α^+ $GranB^+$ cells and TNF-α^+ IFN-γ^+ cells as in (H) (n= 3). I. Representative images of subcutaneous HCC tumors constructed by shCtrl or shHmgb2 Hepa1-6 cell injection in NC and *Hmgb2*-cKO mice (n=5). Tannic acid was given once a day by oral administration. J. Tumor growth curves of HCC subcutaneous tumors as in (I) (n= 5). Data are presented as the mean ± SEM. ns, no significance; *p< 0.05; **p< 0.01; ***p< 0.001; ****p< 0.0001. Student's t test for E and H. Two-way ANOVA test for F and J. OCR, oxygen consumption rate; cKO, *Hmgb2* conditional knockout.

**Celltype**	**Top10 cell markers**									
**NK/T cell**	CCL5	NKG7	IL7R	KLRB1	IFNG	CD69	GNLY	IL32	CD2	GZMA
**Myeloid cell**	C1QA	C1QB	HLA-DRA	CST3	HLA-DPA1	C1QC	CD74	SLC40A1	LGMN	HLA-DRB1
**Endothelial cell**	CCL14	SPARCL1	PLVAP	TM4SF1	IFI27	STC1	GNG11	SPRY1	HES1	IGFBP7
**Neutrophil**	CXCL8	S100A8	G0S2	S100A9	NAMPT	LUCAT1	FPR1	BASP1	C5AR1	PLAUR
**Hepatocyte**	RBP4	ALB	FABP1	AMBP	ORM1	APOA1	HP	VTN	NUPR1	H19
**Fibroblast**	TAGLN	ACTA2	RGS5	COL1A1	CALD1	IGFBP7	COL1A2	COL3A1	MYL9	TPM2
**B cell**	CD79A	IGHM	MS4A1	TNFRSF13C	CD37	BANK1	CD83	LY9	HLA-DQA2	IGHD

Table S1. Top 10 cell markers of different cell types in HCC scRNA-seq data.
